# Back-translating behavioral intervention for autism spectrum disorders to mice with blunted reward restores social abilities

**DOI:** 10.1038/s41398-018-0247-y

**Published:** 2018-09-21

**Authors:** Camille N. Pujol, Lucie P. Pellissier, Céline Clément, Jérôme A. J. Becker, Julie Le Merrer

**Affiliations:** 1 Médecine Translationelle et Neurogénétique, Institut de Génétique et de Biologie Moléculaire et Cellulaire, Inserm U-964, CNRS UMR-7104, Université de Strasbourg, Illkirch, France; 2 Physiologie de la Reproduction et des Comportements, INRA UMR-0085, CNRS UMR-7247, IFCE, Université de Tours, Inserm, Nouzilly, France; 3 Laboratoire Interuniversitaire en Sciences de l’Education et de la Communication, EA 2310, Université de Strasbourg, Strasbourg, France; 4 Present Address: Département de Neurosciences, Institut de Génomique fonctionnelle, Inserm U-661, CNRS UMR 5203, 34094 Montpellier, France

## Abstract

The mu opioid receptor (MOR) plays a critical role in modulating social behavior in humans and animals. Accordingly, MOR null mice display severe alterations in their social repertoire as well as multiple other behavioral deficits, recapitulating core and secondary symptoms of autism spectrum disorder (ASD). Such behavioral profile suggests that MOR dysfunction, and beyond this, altered reward processes may contribute to ASD etiopathology. Interestingly, the only treatments that proved efficacy in relieving core symptoms of ASD, early behavioral intervention programs, rely principally on positive reinforcement to ameliorate behavior. The neurobiological underpinnings of their beneficial effects, however, remain poorly understood. Here we back-translated applied behavior analysis (ABA)-based behavioral interventions to mice lacking the MOR (*Oprm1*^*−/−*^), as a model of autism with blunted reward processing. By associating a positive reinforcement, palatable food reward, to daily encounter with a wild-type congener, we were able to rescue durably social interaction and preference in *Oprm1*^*−/*−^ mice. Along with behavioral improvements, the expression of marker genes of neuronal activity and plasticity as well as genes of the oxytocin/vasopressin system were remarkably normalized in the reward/social circuitry. Our study provides further evidence for a critical involvement of reward processes in driving social behavior and opens new perspectives regarding therapeutic intervention in ASD.

## Introduction

Within the opioid system, the mu opioid receptor (MOR) plays a key role in mediating the rewarding properties of natural and artificial stimuli such as food or drugs of abuse^1,2^. Stemming from the pioneer work of Panksepp and colleagues^3^, evidence from imaging, experimental psychology, and behavioral pharmacology have uncovered how MOR activation also underpins social reward and motivation in humans and animals and, consequently, modulates varieties of social behaviors (sexual and affiliative behaviors, bonding, social play, social exploration)^4–8^. Consistent with this, genetic knockout of MOR (*Oprm1*^*−/−*^) in mice produces severe alterations of their social repertoire, from early life to adult age^9–12^, further demonstrating that MOR is essential for establishing appropriate social behavior. *Oprm1*^*−/−*^ animals were recently proposed to model autism spectrum disorder (ASD)^9,13^, a heterogeneous group of neurodevelopmental diseases whose diagnosis lies on the detection of two types of core symptoms: deficient social reciprocity and communication together with restricted, repetitive patterns of behavior^14,15^. Remarkably, not only MOR null mice recapitulate all these core symptoms but they also display multiple behavioral and physiological abnormalities frequently associated to ASD^7,9,14^, proving unique face validity for this model^16^. Moreover, these animals show several neurobiological landmarks of the disease, such as altered striatal function, decreased activation of the reward circuit in response to social stimuli or reduced oxytocin in the nucleus accumbens^9,11,17–21^ that, together with the identification of ASD patients bearing mutations in the *OPRM1* gene^7^, demonstrate construct validity for this model. Given the key role of MOR in modulating reward, these data suggest, beyond a potential contribution of MOR dysfunction in ASD, that altered reward processes may represent a key mechanism underlying ASD etiopathogeny. Interestingly, a growing body of literature points to reward deficits in patients with ASD^7,18^, in agreement with the social motivation theory of autism proposing that disrupted social interest in these patients would result primarily from early deficits in their motivation for attending, enjoying and prolonging social interactions^22,23^.

If compromised reward processes were to account for behavioral deficits in ASD, a straightforward consequence would be that facilitating these processes in patients should represent an efficient tool to relieve autistic symptoms. To date, available pharmacological treatments for ASD mostly target associated symptoms^24,25^ and evidence-based behavioral interventions remain the only treatments that proved to ameliorate core symptoms^26–29^. Strikingly, the most widely used and longest standing intervention models, Early Intensive Behavioral Intervention (EIBI) programs, based on applied behavioral analysis (ABA), use various behavioral techniques relying on positive reinforcement as levers to shape behavior^30–34^. Concretely, EIBI programs break desirable behaviors (direct eye gaze or speech for example) down into steps and reward success at each step; conversely, these therapies may resort to punishment to discourage behaviors considered as inappropriate (tantrums for example). Directly derived from ABA-based EIBI, novel interventions have recently emerged (such as the Early Start Denver Model, enhanced Milieu teaching, Pivotal Response Treatment, parent-implemented programs), often play-based, in which the intervention is more child-directed and occurs in “naturalistic” environments, to facilitate generalization to the child’s everyday life^35–40^. A common feature of all these interventions remains the use of direct positive reinforcers to promote behavioral improvements, whereas punishment is mostly discarded^29,36,41^. Reinforcers are often edible or tangible items, such as palatable food, preferred by patients with ASD over more social reinforcement, like praise^42,43^. Thus, evidence-based behavioral intervention involves stimulating the reward system to increase the occurrence of appropriate behavior, particularly within the social repertoire, which is in accordance with a reward hypothesis in ASD.

Although they have demonstrated their efficacy, behavioral intervention programs show some limitations. Indeed, these programs are highly demanding to children and their families: they need to be intensive (20–40 h per week) and ought to start at the youngest possible age;^44–46^ however, their outcome remains uncertain. Therapists are seeking experimental evidence and biomarkers to identify key elements predictive of positive results for each patient. In this context, transposing the basic principles of behavioral intervention to mouse models offers a unique opportunity to decipher their neurobiological underpinnings. Here, we back-translated such intervention to *Oprm1*^*−/−*^ mice, as a model of ASD with demonstrated reward deficiency, to assess whether associating positive reinforcement to social experience in these animals could rescue their impaired social abilities (and, eventually, other behavioral deficits), which would argue for a crucial contribution of reward processes in the beneficial effects of behavioral intervention. Moreover, we investigated gene expression in key brain regions for reward processing and social behavior in these animals to better delineate the molecular substrates of these effects.

## Materials and methods

### Animals, housing conditions, and breeding procedures

Male and female *Oprm1*^*+/+*^ and *Oprm1*^*−/−*^ mice^47^ were bred in-house on an identical hybrid background: 50% 129SVPas - 50% C57BL/6 J. *Oprm1*^*+/+*^ and *Oprm1*^*−/−*^ pups were bred from homozygous parents, as we previously showed that parental care has no influence on behavioral phenotype in these animals (cross-fostering experiments, see ref. ^9^). Homozygous parents, however, were bred from heterozygous animals, to prevent genetic derivation. This breeding scheme may have potentiated behavioral deficits in mutant animals by maintaining them together during early postnatal development. Except otherwise stated, animals were group-housed and maintained on a 12 h light/dark cycle (lights on at 7:00 AM) at controlled temperature (21 ± 1 °C); food and water were available ad libitum. Experiments were analyzed blind to genotypes and experimental condition. All experimental procedures were conducted in accordance with the European Communities Council Directive 2010/63/EU and approved by the Comité d’Ethique pour l’Expérimentation Animale de l’ICS et de l’IGBMC (Com’Eth, 2012-033) and Comité d’Ethique en Expérimentation animale Val de Loire (C2EA-19).

### Behavioral experiments

#### Behavioral training protocol

**Fig. 1:**
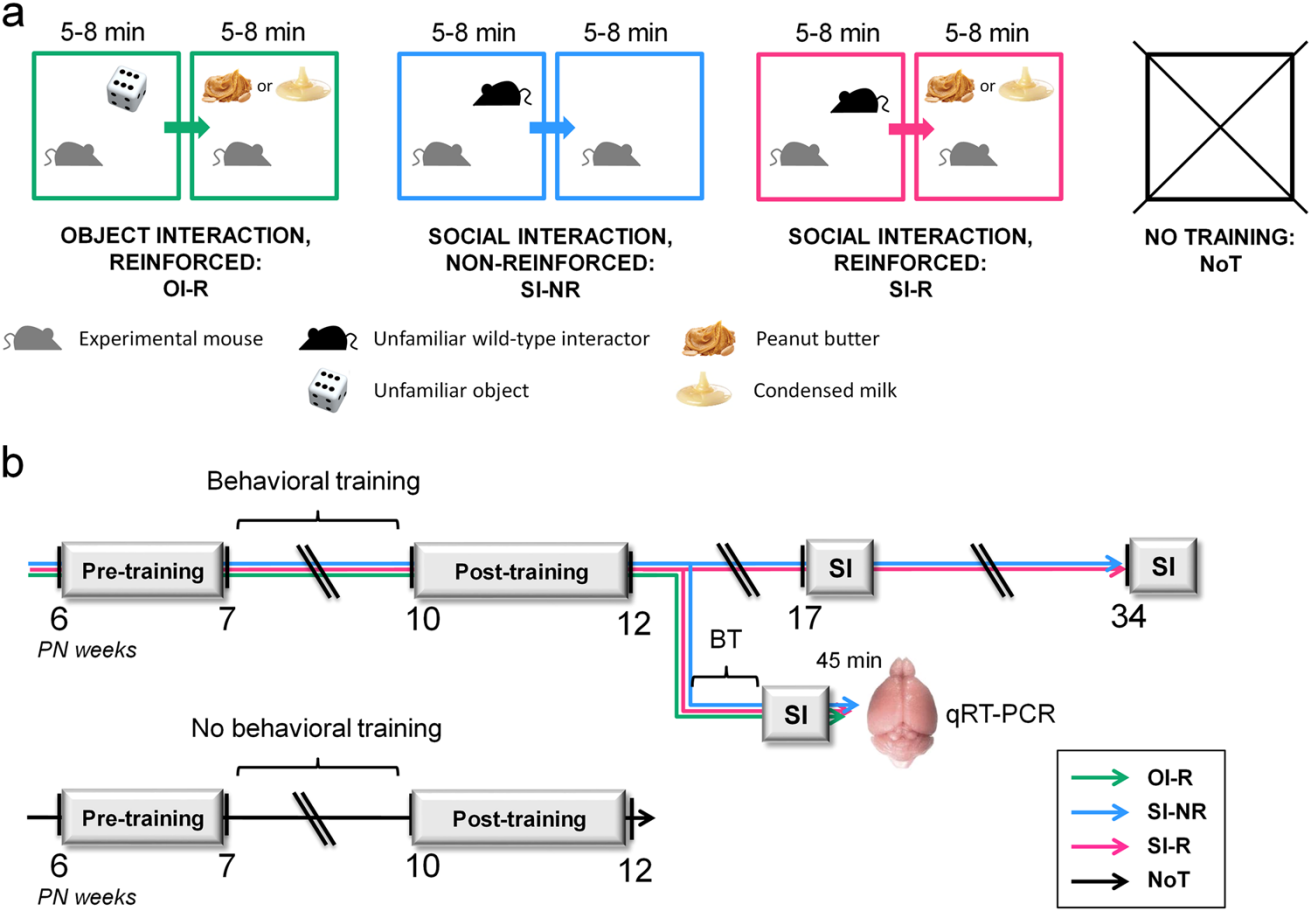
Experimental conditions and time line. **a** *Oprm1*^*+/+*^ and *Oprm1*^*−/−*^ animals were randomly distributed across four experimental conditions: object interaction-reinforced (OI-R: food reward was offered to the animals after exposure to an unfamiliar object), social interaction-nonreinforced (SI-NR: exposure to an unfamiliar wild-type congener was not followed by food presentation), social interaction-reinforced (SI-R: social encounter was followed by the favorite food reinforcer–peanut butter or condensed milk) and no training control (NoT, animals were not manipulated between pre- and post-training behavioral assessments and did not received palatable food). **b** All animals undergone pre-training assays during postnatal (PN) week 6, then behavioral training for 3 weeks (except for the NoT condition) before post-training assessments on PN weeks 10 to 12. Half of the SI-NR and SI-R animals were retested for social interaction after cessation of training on PN weeks 17 and 34. Half of the OI-R, SI-NR, and SI-R animals were trained 3 more days and killed 45 min after the beginning of an ultimate social interaction session (no food reinforcement) for qRT-PCR study

Equivalent numbers of naive male and female animals were used in each group. Female mice were not synchronized for estrous cycle. Experiments started when mice were 6-week old, in an attempt to mimic early intervention conditions. Caregivers in EIBI programs initially define several target behaviors to work on; here we focused on social interaction. Animals were randomly distributed across four experimental conditions before behavioral assays had started: object interaction-reinforced (OI-R), social interaction-nonreinforced (SI-NR), social interaction-reinforced (SI-R), and No Training control (NoT) (see details below and in Fig. 1a). We performed behavioral testing in three steps (time line in Fig. 1b).

##### Pre-training tests

To match clinical conditions^48^, we first evaluated which reinforcer was preferred in our animals between two highly palatable diets: condensed milk or peanut butter. This allowed us to determine the type of palatable food reinforcer to use in further experiments for each animal. Preference was measured in a Y-maze, and exploration pattern during habituation was used to assess perseverative behavior. We evaluated social abilities using the direct social interaction test (postnatal—PN–week 6, see supplemental information for detailed protocols).

##### Behavioral training

Behavioral training started on PN week 7 and lasted 3 weeks. In the SI-R condition (experimental group), mutant and wild-type mice interacted 5 days a week with a wild-type unfamiliar conspecific, different every day, for 5 min during the first 2 weeks, for 8 min during the 3rd week. The interactor was then removed from the arena, whereas the experimental animal remained another 5 (or 8 min) and received a ∼ 3 g unit of its favorite food reward. The amount of food was weighted before and after the test to measure consumption. In a first control group, the SI-NR group, training was performed under the same conditions except that no food reward was available in the arenas following social interaction. In SI-NR and SI-R groups, the time spent in nose contacts and their duration were measured during the course of training to assess the evolution of social behavior (days 2, 4, 8, 12, and 14). During the two last training sessions, some male mice (*Oprm1*^*+/+*^ and *Oprm1*^*−/−*^) from the SI-R group developed aggressive behavior, maybe a territorial response owing to repetitive food presentation in the arena. In a second control group, the OI-R group, food reward was offered to the animals after exposure to an unfamiliar object (different every day) instead of a mouse. The amount of food consumed was measured. Finally, in a no training control group (NoT), the animals were tested on PN weeks 6, 10, and 11 under the same conditions as in the other groups, but were not manipulated during PN weeks 7–9.

##### Post-training tests

Beginning on PN week 10, we performed multiple assays to assess the consequences of behavioral training. Social abilities were explored using the direct social interaction (between unfamiliar animals of the same genotype and experimental group) and three-chamber tests^49^. Stereotyped/perseverative behavior was assessed by scoring motor stereotypies^50^, monitoring alternation in a Y-maze^51^, and assessing anxiety-induced marble burying^52^. Anxiety was evaluated in the novelty suppressed feeding test^53^ (testing order in Figure S1). Detailed behavioral protocols are described in Supplemental information.

In cohorts dedicated to qRT-PCR analysis (half of the OI-R, SI-NR, and SI-R cohorts), animals were submitted to three additional days of behavioral training (week 12), and killed 45 min after the beginning of an additional social interaction session without food presentation (Fig. 1b). To assess the maintenance of training effects over the time, we submitted the other half of the SI-NR and SI-R cohorts to a direct social interaction test on PN weeks 17 and 24 (detailed protocol in Supplemental information).

### Real-time quantitative PCR analysis

Brains were removed and placed into a brain matrix (ASI Instruments, Warren, MI, USA). Caudate putamen (CPu), nucleus accumbens (NAc), central amygdala (CeA), and ventral tegmental area/substancia nigra pars compacta (VTA/SNc) were punched out, whereas prefrontal cortex (PFC) and medial amygdala (MeA) were dissected from 1 mm-thick slices (see Figure S2). Tissues were immediately frozen on dry ice and kept at − 80 °C until use. For each structure of interest, genotype, and condition, samples were prepared from three male and three female mice and processed individually (*n* = 6). RNA was extracted and purified using the MIRNeasy mini-kit (Qiagen, Courtaboeuf, France). cDNA was synthetized using the first-strand Superscript II kit (Invitrogen®, Life Technologies, Saint Thomas, France). qRT-PCR was performed as previously described^9^. Primer sequences are displayed in Table S1.

### Statistical analyses

Statistical analyses were performed using Statistica 9.0 software (StatSoft, Maisons-Alfort, France). All data were initially checked for normality of distribution using Kolmogorov–Smirnov’s test of normality. For all comparisons, values of *p* < 0.05 were considered as significant. Statistical significance in behavioral experiments was assessed using three- to four-way analysis of variance (genotype, gender, condition, and training effects) followed by Newman–Keuls post hoc test. Variance was similar between compared groups. We defined sample size (GPower 3.1) to ensure sufficient statistical power using ANOVA to detect significant effect of our parameters (effect size *f* = 1.80, *α* = 0.05, *σ* = 5, *n* = 8, power = 0.96). Significance of qRT-PCR results was assessed after transformation using a one-sample *t* test, as previously described^9^. A standard principal component analysis (PCA) was performed on behavioral and qRT-PCR data^9^. Loadings for each extracted principal component (PC) are quoted in Table S2. We considered the two first extracted PCs (PC1 and PC2) for schematic representation.

## Results

### Before behavioral training, mice lacking the MOR show perseverative behavior in the Y-maze and severe deficit in social interaction

**Fig. 2:**
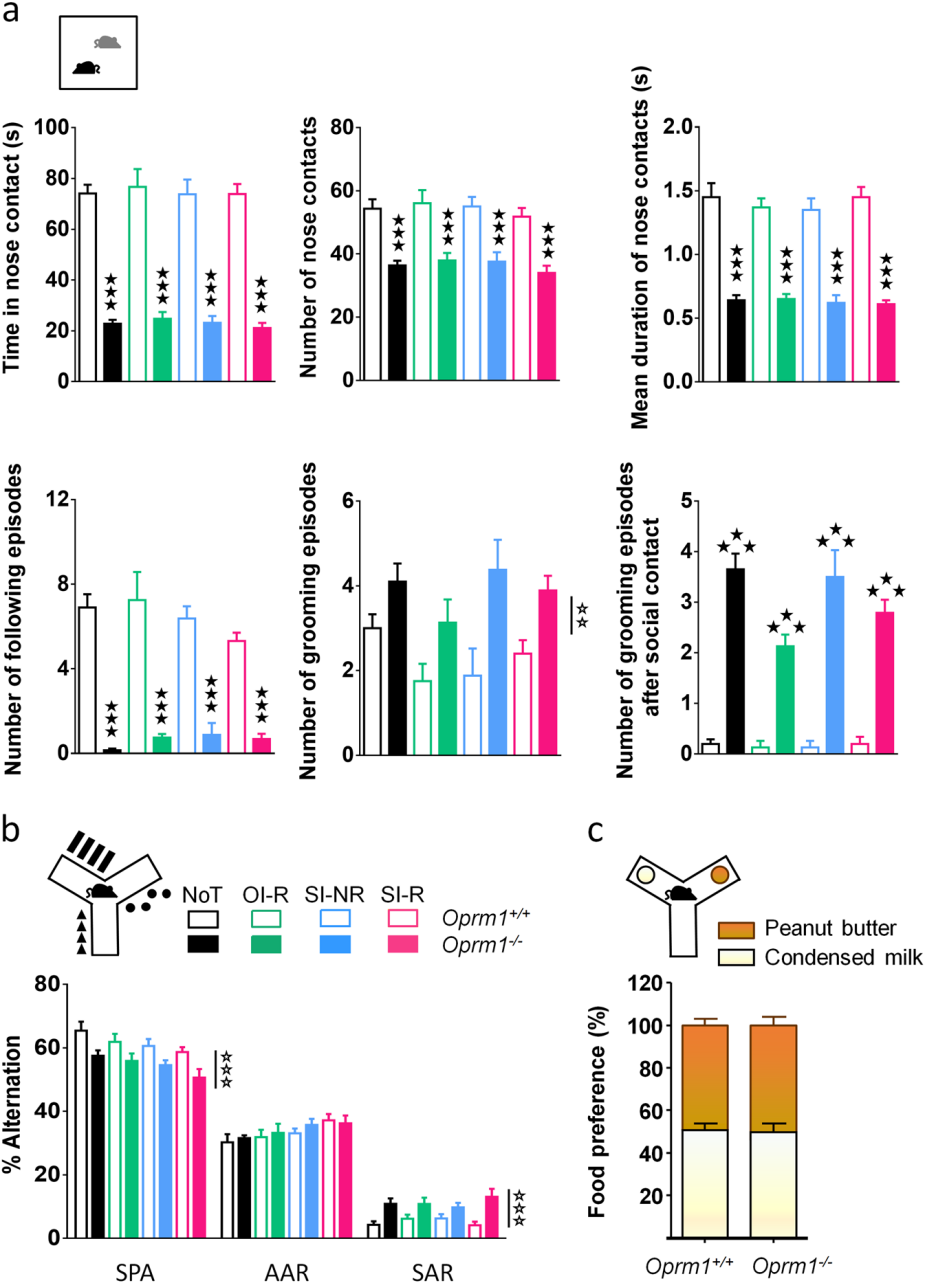
*Oprm1*^*−/−*^ mice display severe social interaction deficit and altered pattern of Y-maze alteration before behavioral training. **a** Before behavioral training, mutant mice (solid bars) in all groups displayed a severe deficit in social interaction when compared with wild-type animals (open bars), as evidenced by decreased time spent in nose contact (NC) with a congener of the same genotype, decreased number of NC and reduced duration of each NC, diminished number of following episodes and increased number of grooming episodes, notably after a social contact. **b** *Oprm1*^*−/−*^ mice showed decreased rates of spontaneous alternation (SPA) and increased rates of same arm returns (SAR) when exploring the Y-maze, independently from training condition. **c** An equal number of mice preferred condensed milk and peanut butter over the other palatable food reinforcer among *Oprm1*^*+/+*^ and *Oprm1*^*−/−*^ animals (OI-R and SI-R groups together). Each animal received its favorite food during training phase. Data are presented as mean ± SEM. Animal numbers per genotype and gender: NoT condition, *n* = 10; OI-R condition, *n* = 8; SI-NR condition, *n* = 8; SI-R condition, *n* = 10–11. Open stars: genotype effect (three-way ANOVA with one repeated measure—type of alternation), solid stars: genotype × condition interaction, comparison with wild-type animals trained under the same condition (three-way ANOVA followed by Newman–Keules *post hoc* test). One symbol: *p* < 0.05, two symbols: *p* < 0.01, three symbols: *p* < 0.001. NoT: no training; OI-R: object interaction-reinforced; SI-NR: social interaction-non-reinforced; SI-R: social interaction-reinforced

We assessed social behavior using the direct social interaction test in mice belonging to the four experimental groups (NoT, OI-R, SI-NR, SI-R) before behavioral intervention. The sex of animals had no significant influence on these parameters. All *Oprm1*^*−/−*^ mice, whatever the group they belonged to, displayed deficient social interaction, as evidenced by decreased time spent in nose contact (NC–genotype effect: *F*_1,125_ = 379.2, *p* < 0.0001), lower number of NC (*F*_1,125_ = 99.2, *p* < 0.0001), decreased mean duration of NC (*F*_1,125_ = 227.0, *p* < 0.0001), and reduced number of following episodes (*F*_1,125_ = 204.3, *p* < 0.0001). Moreover, mutant animals groomed more than wild-type controls (F_1,125_ = 21.3, *p* < 0.01), especially following a social contact (F_1,125_ = 252.3, *p* < 0.0001), a sign of social discomfort^9,54^ (Fig. 2a, statistics in Table S3). We evaluated perseverative behavior before behavioral training by recording the animal’s pattern of exploration in the Y-maze (Fig. 2b). *Oprm1*^*−/−*^ animals from all groups displayed lower rates of spontaneous alternation than *Oprm1*^*+/+*^ animals (genotype: *F*_1,125_ = 26.2, *p* < 0.0001), consistent with an increased number of perseverative same arm returns in mutant mice (*F*_1,125_ = 23.3, *p* < 0.0001). Finally, we measured preference for condensed milk over peanut butter when these reinforcers were made available in the Y-maze. In this test, similar numbers of *Oprm1*^*+/+*^ and *Oprm1*^*−/−*^ animals preferred one over the other reinforcer (percentage of animals preferring condensed milk: *Oprm1*^*+/+*^ 50.69%; *Oprm1*^*−/−*^ 50.13%, Fig. 2c).

### Palatable food intake and social interaction increased during the course of behavioral training in *Oprm1*^*+/+*^ and *Oprm1*^*−/−*^ mice

Over the course of training, *Oprm1*^*+/+*^ and *Oprm1*^*−/−*^ animals increased their intake of palatable food, with mutant mice consuming less (genotype effect: F_1,64_ = 10.3, *p* < 0.01; training effect: *F*_14,386_ = 6.2, *p* < 0.0001) (Figure S3a), especially females (Figure S3b). To assess the evolution of social behavior during training, we monitored (SI-NR and SI-R groups) the time spent in and number of NC, and calculated the mean duration of NC. The three parameters increased over time in both groups (training: time in NC–*F*_4,70_ = 12.1, *p* < 0.0001; number of NC–*F*_4,70_ = 5.4, *p* < 0.001; duration of NC: *F*_4,70_ = 15.4, *p* < 0.0001) with NC in the SI-R group being more numerous than in the SI-NR group (condition: *F*_1,70_ = 6.1, *p* < 0.05) than in the SI-R group (Figure S4a, statistics in Table S4). These two parameters, however, did not differ between genotypes, maybe because wild-type interactors initiated most of the social contacts during these sessions. The mean duration of NC, however, was shorter in *Oprm1*^*−/−*^ animals than in *Oprm1*^*+/+*^ controls, and increased more significantly under the SI-R than the SI-NR condition (genotype: *F*_1,70_ = 13.0, *p* < 0.001; condition: *F*_1,70_ = 10.6, *p* < 0.01; training × genotype: *F*_4,70_ = 2.6, *p* < 0.05). Gender had little influence on these parameters (Figure S4b). Thus, daily exposure to an unfamiliar congener increased the number and duration of NC in mice, especially when this exposure was reinforced with food.

### Behavioral intervention (SI-R) durably relieved social interaction deficit in *Oprm1*^*−/−*^ mice

**Fig. 3:**
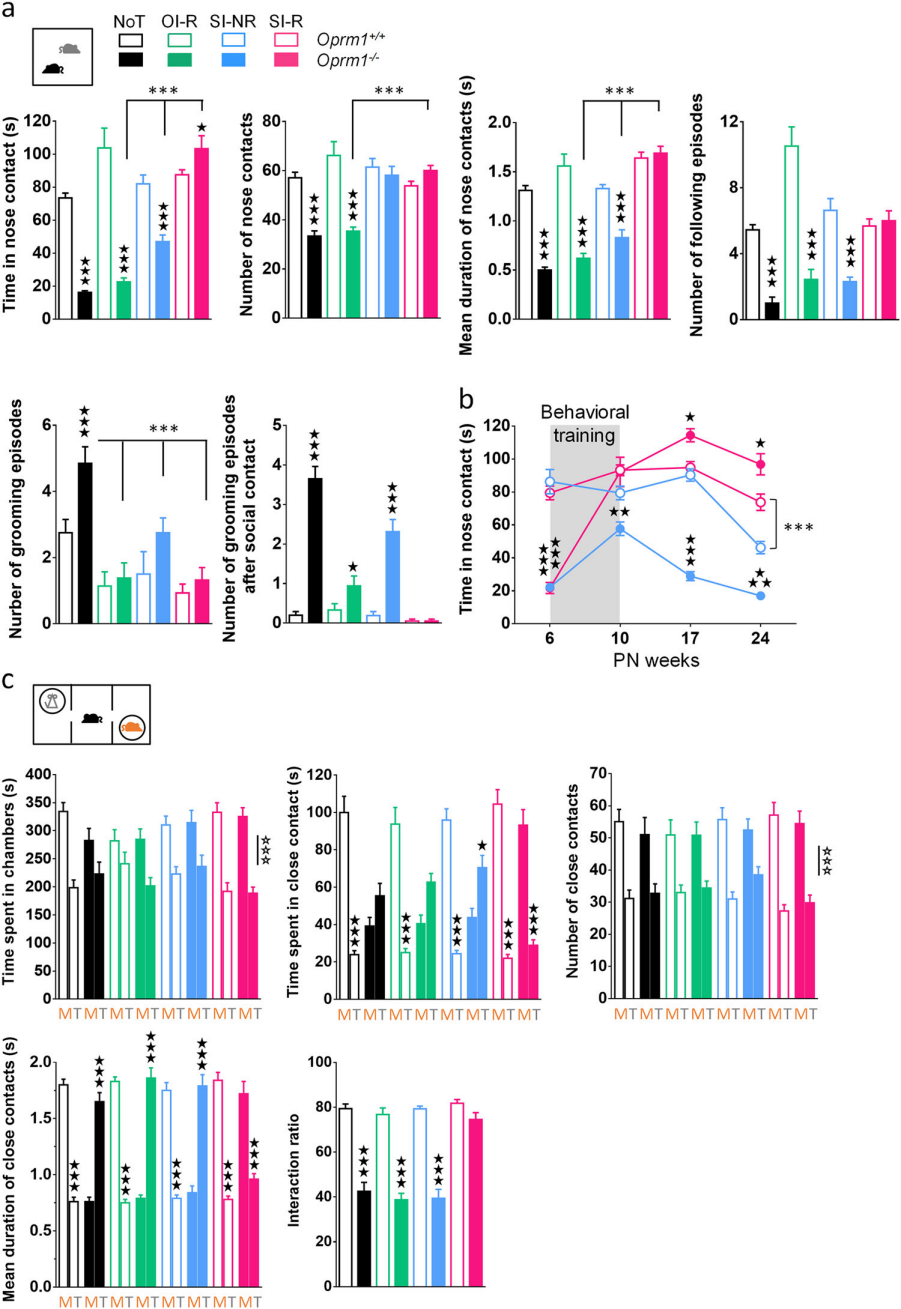
Behavioral intervention (SI-R) durably relieved social interaction deficit in *Oprm1*^*−/−*^ mice. **a** When retested after behavioral training in the direct social interaction test, *Oprm1*^*−/−*^ animals (solid bars) from the NoT and OI-R groups displayed similar severe deficit as before training. Knockout mice from the SI-NR group showed restored number of NC as compared to their *Oprm1*^*+/+*^ controls (open bars) and increased time spent in NC and mean duration of NC when compared with *Oprm1*^*−/−*^ animals of the NoT group, but their number of following episodes and grooming episodes, including after social contact, were unchanged as compared with this control. Finally, all previous interaction parameters were normalized to wild-type levels in *Oprm1*^*−/−*^ mice trained under the SI-R condition. Genotype × condition interaction, solid stars: comparison with wild-type animals treated under the same condition; asterisks: comparison with mutant animals treated under the other conditions (three-way ANOVA followed by Newman–Keules post hoc test). **b** The increase in time spent in NC was maintained up to 24 weeks after complete cessation of behavioral training in knockout mice from the SI-R and not SI-NR group. *Oprm1*^*+/+*^ animals trained under the SI-R condition also maintained greater levels of social interaction over time than their SI-NR counterparts did. See more parameters in Figure S4. Genotype × condition interaction, Solid stars: compared with wild-type animals trained under the same condition; asterisks: *Oprm1*^*+/+*^ SI-R compared with *Oprm1*^*+/+*^ SI-NR (four-way ANOVA with week of testing as a repeated measure, followed by Newman–Keules post hoc test). **c** In the three-chamber test, *Oprm1*^*+/+*^ and *Oprm1*^*−/−*^ mice from all experimental groups spent more time in the chamber with the mouse and made more contacts with the mouse than with the toy. Wild-type mice, however, spent significantly more time in close contact with the mouse than with the toy, each contact lasting longer with the mouse than with the toy. In contrast, *Oprm1*^*−/−*^ mice made longer contacts with the toy and thus displayed no preference for spending time in contact with the mouse, except in the SI-R group. Consequently, preference ratio was markedly reduced in mutant mice as compared with wild-type, except for *Oprm1*^*−/−*^ mice trained under the SI-R condition, that displayed similar social preference as *Oprm1*^*+/+*^ animals. Solid stars: genotype × condition interaction, comparison with wild-type animals treated under the same condition; open stars: genotype effect (four-way ANOVA with one repeated measure: stimulus—mouse versus toy followed by Newman–Keules post hoc test). Data are presented as mean ± SEM. Animal numbers per genotype and gender in **a** and **c**: NoT condition, *n* = 10; OI-R condition, *n* = 7–8; SI-NR condition, *n* = 8; SI-R condition, *n* = 10–11. Animal numbers per genotype and gender in **b**: SI-NR condition: *n* = 4; SI-R condition: *n* = 4–6. One symbol *p* < 0.05, two symbols *p* < 0.01, three symbols *p* < 0.001. M: mouse; NoT: no training; OI-R: object interaction-reinforced; SI-NR: social interaction-non-reinforced; SI-R: social interaction-reinforced; T: toy

We assessed social behavior in mice submitted to the four different conditions of behavioral training using two assays: the direct social interaction test and the three-chamber social preference test. In the former, *Oprm1*^*−/−*^ mice from the NoT and OI-R groups still displayed a marked deficit in social interaction as compared to their wild-type controls, although the number of grooming episodes, including these occurring after social contact, was reduced in OI-R versus NoT mutant animals. Knockout mice from the SI-NR group showed restored number of NC as compared with their *Oprm1*^*+/+*^ controls, increased time spent in NC and mean duration of NC and decreased total number of grooming episodes when compared with knockout animals of the NoT group. However, the number of following episodes and grooming episodes after social contact were unchanged as compared with this control. Finally, all interaction parameters were normalized to wild-type levels in male and female *Oprm1*^*−/−*^ mice trained under the SI-R condition (genotype × treatment: time in NC–*F*_3,125_ = 28.0, *p* < 0.0001; number of NC–*F*_3,125_ = 18.1, *p* < 0.0001; duration of NC–*F*_3,125_ = 24.6, *p* < 0.0001; following—*F*_3,125_ = 18.3, *p* < 0.0001; number of grooming episodes—*F*_3,125_ = 13.0, *p* < 0.01; grooming after social contact—F_3,125_ = 40.7, *p* < 0.0001; Fig. 3a, more parameters and sex effects in Figure S5, statistics in Table S5). We further tested social interaction in half of the SI-NR and SI-R cohorts 7 and 14 weeks after cessation of training and observed a persistence of the beneficial effects of behavioral training in mutants from the SI-R but not the SI-NR group, as illustrated by maintained normalization of the time in NC. Interestingly, 14 weeks after cessation of training time in NC was also higher in *Oprm1*^*+/+*^ mice from the SI-R group compared with SI-NR (training × genotype × condition: *F*_3,84_ = 13.6, *p* < 0.0001) (Fig. 3b, more parameters and sex effects in Figure S6, statistics in Table S6).

We further assessed social behavior across experimental groups using the three-chamber test after behavioral training (Fig. 3c and S7). In this test, male and female *Oprm1*^*−/−*^ mice from the NoT, OI-R, and SI-NR groups displayed a significant deficit in social preference, as shown by absent preference for time spent in close contact with the mouse over the toy (stimulus × genotype × condition: *F*_3,123_ = 9.7, *p* < 0.0001), longer duration of close contacts with the toy versus the mouse (stimulus × genotype × condition: *F*_3,123_ = 41.4, *p* < 0.0001) and, consequently, diminished preference ratios (genotype × condition: F_3,123_ = 15.5, *p* < 0.0001) when compared with respective *Oprm1*^*+/+*^ controls. In contrast, mutant animals trained under the SI-R condition preferred to spend more time in close contact with the mouse over the toy, displayed longer close contacts with the mouse and accordingly showed a fully restored preference ratio as compared with *Oprm1*^*+/+*^ animals from the same group (statistics in Table S5). Thus, beneficial effects of behavioral intervention in *Oprm1*^*−/−*^ mice trained under the SI-R condition generalized to social preference measured in the three-chamber test.

### Beneficial effects of behavioral intervention in mutant mice were limited to the social repertoire

**Fig. 4:**
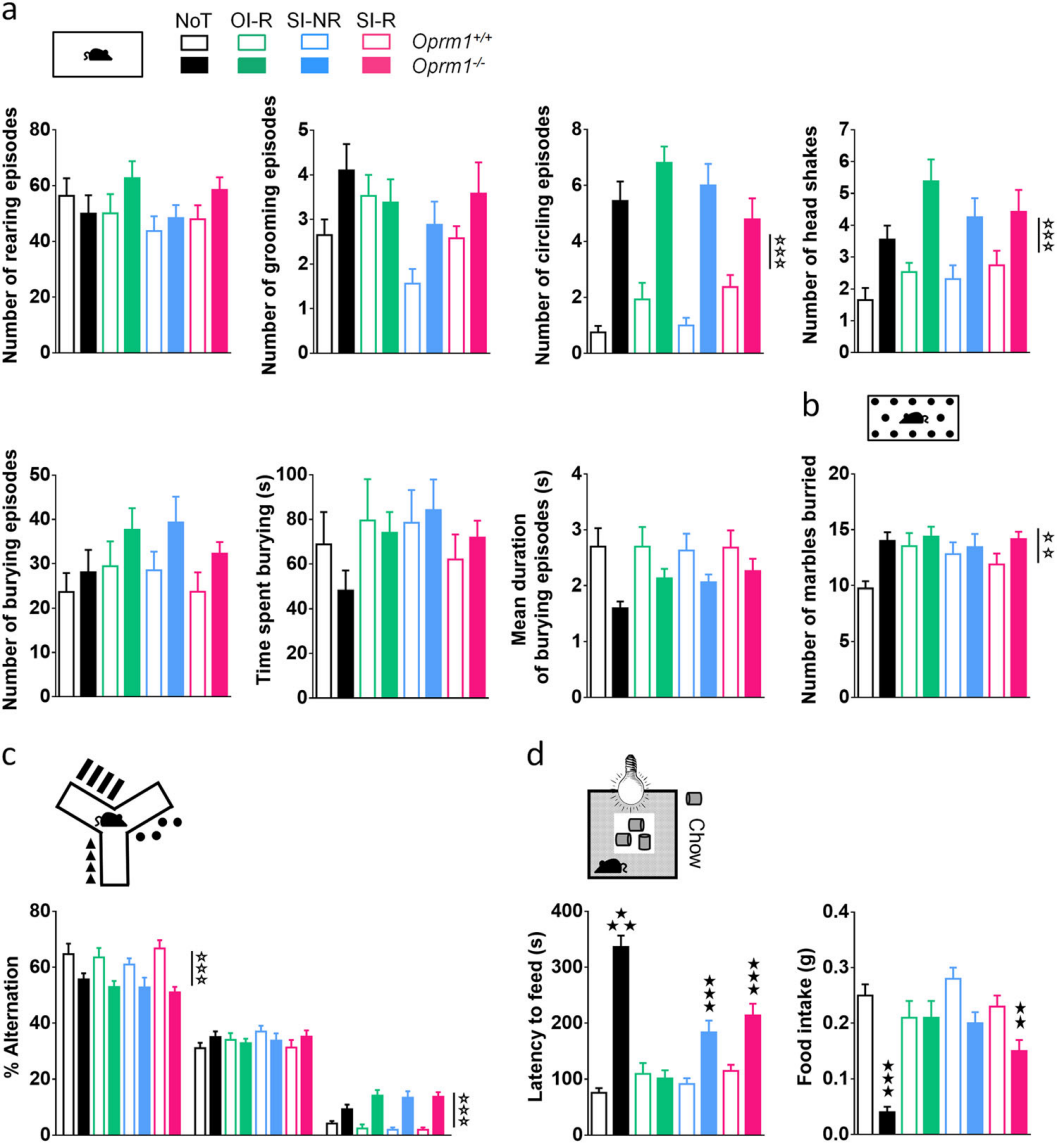
Behavioral intervention (SI-R) did not modify non-social (off-target) behaviors. **a** When tested after behavioral training, *Oprm1*^*−/−*^ mice (solid bars), compared with wild-type animals (open bars), showed increased number of grooming and circling episodes, more frequent head shakes and shorter burying episodes. **b** Mutant animals buried more marbles in the marble burying test and **c** showed decreased rates of spontaneous alternation (SPA) and increased rates of same arm returns (SAR) when exploring a Y-maze, independently from training condition. **d** In the novelty suppressed feeding test, the latency to feed of mutant mice was reduced after training in the SI-NR and SI-R conditions and normalized to *Oprm1*^*+/+*^ levels in the OI-R group. Data are presented as mean ± SEM. Animal numbers per genotype and gender: NoT condition, *n* = 10; OI-R condition, *n* = 7–8; SI-NR condition, *n* = 8; SI-R condition, *n* = 10–11. Open stars: genotype effect; solid stars: genotype × condition interaction (three-way ANOVA followed by Newman–Keules post hoc test). Two stars: *p* < 0.01, three stars: p < 0.001. NoT: no training; OI-R: object interaction-reinforced; SI-NR: social interaction-non-reinforced; SI-R: social interaction-reinforced

We assessed whether the effects of behavioral training on social behavior would extend to other, non-social, behavioral deficits in *Oprm1*^*−/−*^ mice. We measured spontaneous motor stereotypies in all groups after training. Mutant mice displayed more frequent grooming (genotype: *F*_1,125_ = 8.1, *p* < 0.01), burying (*F*_1,125_ = 5.7, *p* > 0.05), circling (*F*_1,125_ = 113.3, *p* < 0.0001), and shakes (*F*_1,125_ = 30.5, *p* < 0.0001) than wild-type animals independently from the experimental condition, except for grooming that was lower in the SI-NR group (condition: *F*_3,125_ = 2.9, *p* < 0.05). Also, burying episodes were shorter in *Oprm1*^*−/−*^ compared with *Oprm1*^*+/+*^ animals through all conditions (genotype: *F*_1,125_ = 12.0, *p* < 0.001) (Fig. 4a). In the marble burying test, knockout mice buried more marbles than wild-type animals, with no significant effect of the experimental condition (genotype: *F*_1,125_ = 9.0, *p* < 0.05) (Fig. 4b). Similarly, in the Y-maze test, behavioral training failed to reduce the number of perseverative same arm entries (genotype: *F*_1,125_ = 104.1, *p* < 0.0001) and restore spontaneous alternation rates (*F*_1,125_ = 50.1, *p* < 0.0001) in mutant mice (Fig. 4c). Finally, we assessed anxiety levels following behavioral training using the novelty suppressed feeding test. In this test, *Oprm1*^*−/−*^ mice took longer to eat on the food pellets, with mice from the OI-R, SI-NR, and SI-R condition eating faster than NoT animals. Under the OI-R condition, the latency to feed of *Oprm1*^*−/−*^ mice was normalized to wild-type levels (genotype × condition: *F*_3,124_ = 24.3, *p* < 0.0001) (Fig. 4d). This latency was also returned to wild-type levels in male but not female mutant animals trained under the SI-NR and SI-R conditions (gender: *F*_1,124_ = 6.8, *p* < 0.01; gender × genotype × condition: *F*_3,124_ = 2.7, *p* < 0.05) (Figure S5). As regards food intake, *Oprm1*^*−/−*^ mice ate less than *Oprm1*^*+/+*^ animals when back in home cage and so did control mice (NoT) compared with other conditions. Mutant mice ate as much as wild-type animals under the OI-R and SI-NR conditions (genotype × condition: *F*_3,124_ = 9.0, *p* < 0.0001; statistics in Table S5). Thus behavioral training reduced anxiety levels in male and female *Oprm1*^*−/−*^ mice in the OI-R group. Altogether, these data indicate that such training had limited impact on off-target, non-social, behaviors.

### Behavioral intervention normalized gene expression in the reward circuit of *Oprm1*^*−/−*^ mice

**Fig. 5:**
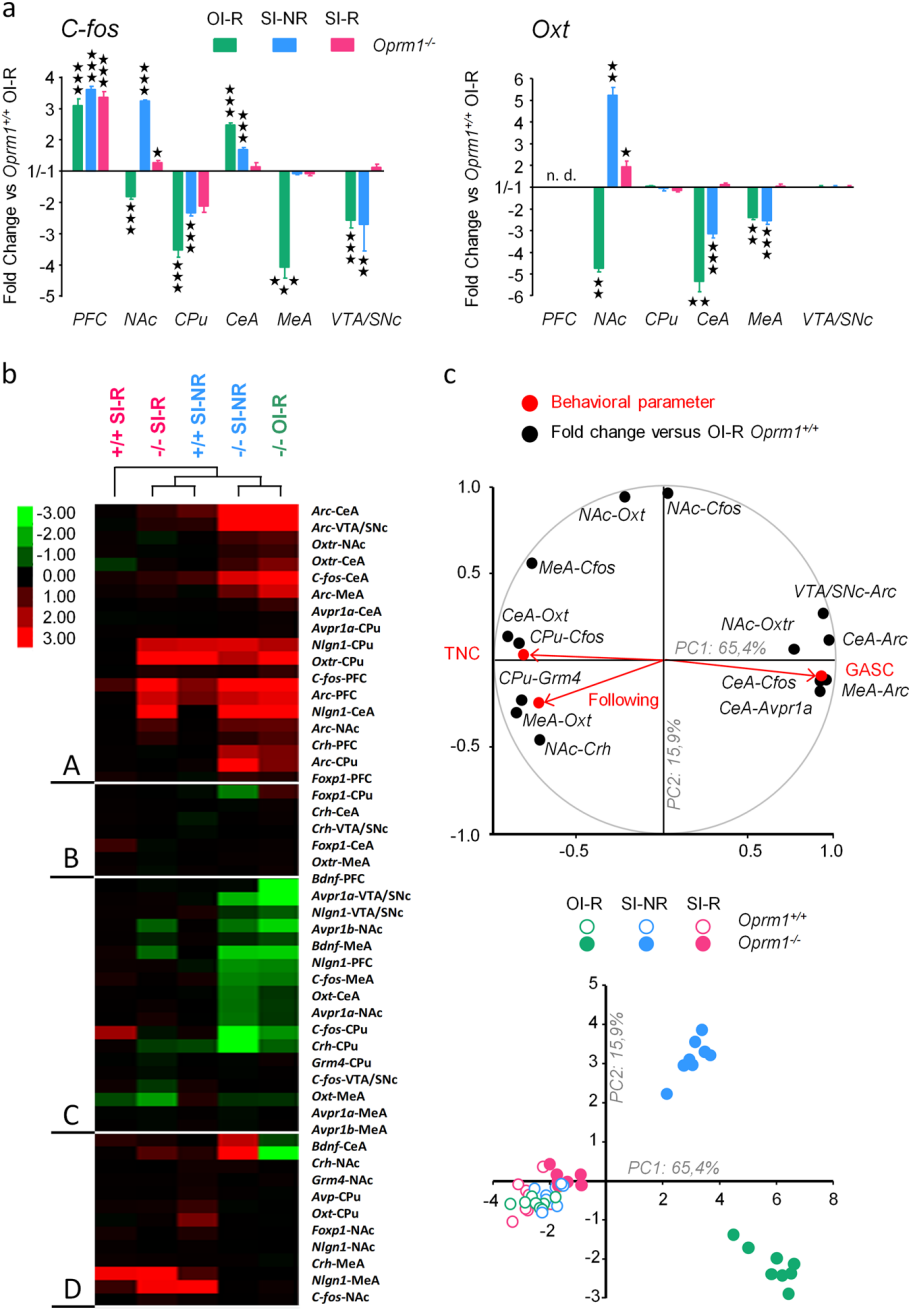
Behavioral intervention normalized plasticity marker and oxytocin/vasopressin gene expression in *Oprm1*^*−/−*^ mice. **a** In *Oprm1*^*−/−*^ mice from the OI-R group, *C-fos* expression was decreased in the NAc, CPu, MeA, and VTA/SNc but increased in the PFC and CeA when compared with *Oprm1*^*+/+*^ trained under the same condition (used as a reference). Training under the SI-NR condition restored *C-fos* transcription levels totally in the NAc (even increased) and MeA and partially in the CPu and CeA, but left these levels unchanged in the PFC and VTA/SNc. SI-R training normalized C-fos mRNA levels in all brain regions but the PFC of these mice. *Oprm1*^*−/−*^ mice from the OI-R group also displayed decreased levels of *Oxt* transcripts in the NAc, CeA, and MeA. When trained under the SI-NR condition, these animals exhibited restored levels of *Oxt* mRNA in the NAc (even increased), partial increase in the CeA and no change in the MeA; mutant mice trained under the SI-R condition instead showed normalized levels of *Oxt* mRNA in all these regions. Gene expression in wild-type animals from the SI-NR and SI-R are not presented for simplification (see Table S7). **b** Hierarchical clustering organized gene expression data in four main clusters. Cluster (A) grouped a majority of genes with upregulated expression in *Oprm1*^*−/−*^ mice trained under the SI-NR and OI-R conditions compared with wild-type (SI-NR or SI-R) or mutant SI-R trained animals. Conversely, cluster (C) brought together genes with downregulated expression in OI-R and SI-NR trained mutant mice. Most of these downregulations were detected in the striatum and amygdala. Cluster (B) gathered genes whose expression was not significantly regulated in these regions. Gene expression in cluster (D) was either oppositely regulated between mutant mice trained under the SI-NR and OI-R conditions, not regulated in these groups, whereas increased in other groups or not regulated. This analysis revealed that gene expression pattern in *Oprm1*^*−/−*^ mice trained under the SI-R condition was more similar to that of wild-type animals (SI-NR or SI-R groups) than that of *Oprm1*^*−/−*^ mice trained under the SI-NR or OI-R conditions. **c** A PCA performed on a selection of three social interaction parameters and qRT-PCR results for 14 genes unraveled the opposition between prosocial parameters (time in social contact, following) and a social avoidance parameter (grooming after social contact) along PC1 (variables’ space, top panel). *C-fos* expression in the CPu, *Oxt* expression in the MeA and CeA, *Crh* mRNA levels in the NAc and *Grm4* levels in the CPu cluster with the former whereas *Arc* in the VTA, CeA, and MeA, *Oxtr* in the NAc, *C-fos* in the CeA and *Avpr1a* in the CeA cluster with the latter. Projection in the subjects’ space (down panel) clearly dissociated *Oprm1*^*−/−*^ individuals of the OI-R and SI-NR groups from *Oprm1*^*+/+*^ mice along PC1, with PC2 axis segregating SI-NR from OI-R populations. Remarkably, individual *Oprm1*^*−/−*^ mice from the SI-R group clustered with *Oprm1*^*+/+*^ animals, showing that, when focusing on this particular set of behavioral and gene expression data, SI-R training normalized *Oprm1*^*−/−*^ features. Gene expression data are expressed as fold change versus the *Oprm1*^*+/+*^ OI-R group (median ± SEM). Behavioral data are presented as mean ± SEM. Animal numbers per genotype, gender, and training condition: *n* = 4. One star *p* < 0.05, two stars *p* < 0.01, three stars *p* < 0.001. CeA: central nucleus of the amygdala; CPu: caudate putamen; MeA: medial nucleus of the amygdala; NAc: nucleus accumbens; OI-R: object interaction-reinforced; PFC: prefrontal cortex; SI-NR: social interaction-non-reinforced; SI-R: social interaction-reinforced; VTA/SNc: ventral tegmental area/substantia nigra pars compacta. See social interaction parameters before qRT-PCR experiment in Figure S8; principal components extracted in PCA analysis are displayed in Table S2

To identify molecular correlates of the behavioral improvements detected after training, we assessed in OI-R, SI-NR, and SI-R mice the expression of 12 genes across six brain regions known to play a key role in reward and social behavior: PFC, CPu, NAc, CeA, MeA, and VTA/SNc. We focused on immediate early genes and markers of plasticity (*C-fos*, *Arc/Arg3.1*, *Bdnf*), genes of the oxytocin/vasopressin system (*Oxt*, *Avp*, *Oxtr*, *Avpr1a*, *Avpr1b*) because of their key role in social behavior and evidences of altered function in *Oprm1*^*−/−*^ mice^9,11^, autism gene candidates (*Nlgn1, Foxp1, Crh*) whose expression was dysregulated in this model^9^ and finally *Grm4*, encoding mGluR4 receptors whose activation relieves autistic symptoms^9^. Fold changes were calculated using expression in *Oprm1*^*+/+*^ mice from the OI-R group as a reference; they are presented in Table S7. Among tested genes, *C-fos* and *Oxt* particularly retained our attention (Fig. 5a). We detected a decrease in *C-fos* expression, a marker gene for neuronal activation, in most regions of the social/reward circuit (NAc, CPu, MeA, and VTA/SNc) of *Oprm1*^*−/−*^ mice trained under the OI-R condition, except for the PFC and CeA where this expression was increased. Repeated interaction with a conspecific under the SI-NR condition restored *C-fos* transcription levels totally in the NAc and MeA and partially in the CPu and CeA, but left these levels unchanged in the PFC and VTA/SNc. SI-R training normalized C-fos mRNA levels in all brain regions but the PFC of mutant mice. As regards *Oxt* expression, we detected reduced levels of transcripts in the NAc, CeA, and MeA of *Oprm1*^*−/−*^ mice from the OI-R group. Mutant mice trained under the SI-NR condition exhibited restored levels of *Oxt* mRNA in the NAc (even increased), partial increase in the CeA and no change in the MeA; the same animals trained under the SI-R condition exhibited normalized levels of *Oxt* mRNA in all these regions. Thus behavioral intervention rescued deregulated expression of *C-fos* and *Oxt* in *Oprm1*^*−/−*^ animals, whatever their direction (up- or downregulation). Training under the SI-NR paradigm ameliorated, but only partially, this expression.

We performed a cluster analysis of all qRT-PCR data to visualize patterns of gene expression depending on genotype and experimental condition (Fig. 5b). Hierarchical clustering organized gene expression data in four main clusters. Cluster (A) grouped a majority of genes with upregulated expression in *Oprm1*^*−/−*^ mice trained under the SI-NR and OI-R conditions compared with wild-type (SI-NR or SI-R) or mutant SI-R trained animals. Remarkable genes in this cluster were *Arc*, markedly upregulated in all regions studied for OI-R and SI-NR trained *Oprm1*^*−/−*^ mice, *Oxtr* (coding oxytocin receptors), upregulated in the amygdala of mice from the OI-R and SI-NR groups and *Crh*, upregulated in the PFC, CPu, and VTA/SNc of mutant animals. Conversely, cluster (C) brought together genes with downregulated expression in OI-R and SI-NR trained mutant mice. Most of these downregulations were detected in the striatum and amygdala (such as *C-fos, Oxt, Crh, Bdnf, Grm4, Avpr1a*, or *Avpr1b*). Cluster (B) gathered genes whose expression was not significantly regulated in these regions. Gene expression in cluster (D) was either oppositely regulated between mutant mice trained under the SI-NR and OI-R conditions (*C-fos* and *Oxt* in the NAc), not regulated in these groups while increased in other groups (*Avp* in the NAc, *Bdnf* in the NAc and CPu) or not regulated. Globally, this analysis unraveled that gene expression pattern in knockout animals trained under the SI-R condition was more similar to that of wild-type animals (SI-NR or SI-R groups) than that of *Oprm1*^*−/−*^ mice trained under the SI-NR or OI-R conditions.

Finally, we performed a PCA including social interaction parameters and gene expression data in different regions to assess correlations between these different outputs (Fig. 5c and S8, Tables S2 and S7). We selected three behavioral parameters and 14 qRT-PCR results that together accounted for 81.5% of the variance in this sample (variables’ space, Fig. 5c, top). We found that the time spent in NC and number of following episodes, two measures of prosocial behavior, clustered together and with *C-fos* expression in the CPu, *Oxt* expression in the MeA and CeA, *Crh* mRNA levels in the NAc and *Grm4* levels in the CPu. This first cluster was opposed along the first principal component (PC1) to a second centered on the number of grooming episodes occurring immediately after social contact, an index of social avoidance, and gathering expression of *Arc* in the VTA, CeA, and MeA, *Oxtr* in the NAc, *C-fos* in the CeA and *Avpr1a* in the CeA. *Oxt* and *C-fos* expression in the NAc and *C-fos* levels in the MeA correlated mostly with PC2. Projection in the subjects’ space (Fig. 5c, bottom) dissociated *Oprm1*^*−/−*^ individuals of the OI-R and SI-NR groups from *Oprm1*^*+/+*^ mice, with PC2 axis segregating SI-NR from OI-R populations. Remarkably, and consistent with the results of clustering analysis, data from individual *Oprm1*^*−/−*^ mice from the SI-R group clustered with those of *Oprm1*^*+/+*^ animals (OI-R, SI-NR, and SI-R condition), showing that, when focusing on this particular set of behavioral and gene expression data, SI-R training (behavioral intervention) normalized *Oprm1*^*−/−*^ features.

## Discussion

To our knowledge, this is the first report of a successful back-translation of ABA-based EIBI to a murine model of autism. We used mice lacking MOR, which display blunted reward processing^1,9,13^, a predictor of positive outcome for behavioral intervention in children with ASD^55,56^, and demonstrated that associating a positive reinforcement to daily encounters with an unfamiliar wild-type congener is sufficient to durably restore direct social interaction, social preference in the three-chamber test and gene expression in the reward/social circuit of these animals, similarly in males and females.

Transposing complex intervention programs such as ABA-based EIBI to a rodent model is a true challenge, and our experimental paradigm shows some methodological limitations. First, EIBI utilize various elaborated paradigms to modify target behaviors in patients with autism^30–34^, whereas here we used appetitive conditioning solely. However, most of the techniques used in behavioral interventions involve positive reinforcement and thus stimulate the reward system, as we did in the present experiments. As a positive reinforcer, we used food reward, as commonly done in the first stages of EIBI^43^. Of note, mutant animals, especially females, consumed less-palatable food than *Oprm1*^*+/+*^ mice during the course of training, suggesting reduced motivation for food, as previously described^57^. These results are in agreement with a key role of MOR in mediating the hedonic “liking” for food^58,59^. *Oprm1*^*−/−*^ mice, however, can learn an operant task to obtain food^57,60^. In our study, they modified their behavior when exposed to palatable food, further showing that they can use food reward as a reinforcer. Second, EIBI therapists usually reinforce each occurrence of the target behavior immediately (fixed ratio 1) and depending on its quality (differential reinforcement)^61,62^. Here we instead used a trace conditioning procedure, palatable food being presented with a delay after social encounter, and provided the same amount of food whatever the quality of previous social interaction. Although this paradigm likely made the association between food reward and social experience more difficult to acquire, improved social interaction in *Oprm1*^*−/−*^ animals from the SI-R group after training demonstrates that these animals indeed made such association. Importantly, food reward was not able to rescue social interaction when not associated to social experience (OI-R condition), showing that behavioral improvements under the SI-R condition were not due to reward exposure only. Moreover, experiencing social encounter with a wild-type stranger every day without receiving a reward (SI-NR) partially restored social interaction in mutants, in line with clinical reports showing lower symptom severity in patients with ASD interacting with typical peers, notably at school^63^. These improvements, though, rapidly faded when daily encounters ceased. In contrast, social enrichment produced partial but persistent beneficial effects on social abilities in *Oprm1*^*−/−*^ mice when provided from neonatal age^64^. Earlier training under the SI-NR condition may thus be required to obtain stable social improvements in these animals. Despite the above limitations, our behavioral intervention (SI-R) paradigm successfully allowed us to modify social behavior in *Oprm1*^*−/−*^ mice by using positive reinforcement, in agreement with our primary hypothesis.

Remarkably, our transposition of behavioral intervention successfully reproduced several key features of EIBI in MOR null mice. As shown in the clinics^29,45^, the effects of intervention were long lasting: they were still detectable 14 weeks after complete cessation of intervention. Such long-lasting beneficial effects of SI-NR training were observed independently from the sex of the animals. Clinical studies reporting positive effects of EIBI include both male and female patients; due to limited numbers of girls, however, these studies do not comment on sex effects^29,45,65^. Interestingly, behavioral intervention (SI-R condition) also showed beneficial effects in *Oprm1*^*+/+*^ mice, by preserving high levels of social interaction in aging (24 weeks) animals. This result further demonstrates the key role played by reward in sustaining social behavior^7,66^. Furthermore, *Oprm1*^*−/−*^ mice of the SI-R group showed only modest improvements in off-target, non-social, behaviors, namely motor stereotypies or anxiety. Based on clinical data, we could have expected some reduction of stereotyped behaviors;^29^ however, our paradigm may not have been comprehensive and/or intensive enough to verify this. Of note, knockout mice from the OI-R group showed reduced anxiety in the novelty suppressed feeding test, likely a consequence of associating daily novel object exploration with a reward: we thus involuntarily trained these animals to display less anxiety in this particular context.

Last, behavioral intervention modified neural substrates in *Oprm1*^*−/−*^ mice, as shown by regulated gene expression. We analyzed the expression pattern of a small collection of genes across six brain regions within the reward/social circuit and revealed that back-translating EIBI in mice was able to normalize this pattern, in correlation with behavioral markers of social abilities. As regards neuronal activity, we detected reduced *C-fos* mRNA level following social interaction in the reward/social circuit (NAc, CPu, MeA, and VTA/SNc), as opposed to increased expression in executive control and anxiety-related PFC and CeA, in knockout mice from the OI-R group, extending previous observations^9^. These results likely reflect modified connectivity within reward/aversion pathways in these animals^17^ and are coherent with their reduced reward sensitivity, social interest, and increased anxiety^9,13^. They also match imaging data in ASD patients showing decreased activity in the reward circuit in response to social stimuli^7,67,68^ and increased activity in the amygdala under anxiogenic social conditions^69^. Remarkably, SI-R training normalized *C-fos* expression in all these regions but the PFC. This result suggests that SI-R paradigm was able to reattribute rewarding properties to social interaction and to decrease anxiety in this context. Now regarding plasticity gene markers, we report for the first time excessive widespread *Arc* expression in the brain of *Oprm1*^*−/−*^ mice following social interaction. In the CeA, MeA, and VTA/SNc, increased *Arc* levels were tightly correlated with a behavioral marker of social avoidance, grooming after social contact. *Arc* codes for activity-regulated cytoskeletal-associated protein (Arc), involved in regulating synaptic plasticity, cellular signaling, glutamate neurotransmission, and spine growth^70,71^. Intriguingly, levels of Arc protein were found increased in the brains of two other mouse models of ASD^72,73^ as well as in the blood of patients with autism^74^. Deficient Arc expression, however, may also be detrimental to social behavior, as shown by impaired sociability and schizophrenia-related phenotype in mice with invalidated *Arc* gene^75^ as well as genetic association between mutations in *Arc* and schizophrenia in humans^76–78^. Together, these data support the hypothesis of a functional connection between Arc and neurodevelopmental diseases with impaired social abilities, namely autism and schizophrenia^79^. Remarkably, SI-R but not SI-NR trained *Oprm1*^*−/−*^ mice displayed normalized *Arc* expression in the CeA, MeA, and VTA/SNc, indicating that plastic events occurred in these regions that likely contributed to improve social abilities. Still related to plasticity, SI-R training induced or restored the expression of *Bdnf* in the NAc and CeA, respectively, of *Oprm1*^*−/−*^ mice. Together, these results match data from imaging studies showing EIBI-induced brain plasticity in autistic patients^80^, specifically in the reward circuit for subjects who display initial hypoactivation in these regions^55^.

Now focusing on genes from the oxytocin/vasopressin system, behavioral training had also major effects on their expression in *Oprm1*^*−/−*^ mice. First, we confirmed our previous observation of decreased levels of *Oxt* mRNA, coding for oxytocin, in the NAc of *Oprm1*^*−/−*^ mice (OI-R group)^9^ and extended it to the CeA and MeA, where they were correlated with prosocial behavioral parameters. Accordingly, transcripts for the oxytocin receptor (*Oxtr*) were found increased in the same regions, matching binding data^11^. The detection of *Oxt* transcripts (as well as *Avp* mRNAs, coding for vasopressin) in brain regions outside the hypothalamus, where oxytocinergic (and vasopressinergic) neurons are localized^81^, might appear surprising: it implies that these mRNAs where transported to projection sites of oxytocinergic neurons. In agreement with this, *Oxt* mRNAs have recently been evidenced in distal projections of human stem cell-derived neurons^82^, suggesting that local transcription may play an important role in oxytocin (and possibly vasopressin) neurotransmission. On the vasopressin side, expression of *Avpr1a*, coding V1_A_R receptors, in mutants, was decreased in the NAc and MeA but increased in the CeA. All these data point to major alterations of the oxytocin/vasopressin system in MOR null mice that likely contributed to their autistic-like phenotype, as shown in other animal models and in patients^83–86^. Singularly, behavioral intervention brought oxytocin/vasopressin gene expression back to wild-type levels or even higher for *Oxt* and *Avp* in the NAc. Thus, beneficial effects of intervention on social behavior in mutants likely involved restored oxytocin/vasopressin function, a key neurobiological substrate for social reward^87^. Pharmacological manipulations of this system, using either oxytocin receptor agonists (including oxytocin) or V1_A_R receptor antagonists, can restore social abilities in animal models of ASD^85^, including *Oprm1*^*−/−*^ mice^11^ and in patients with autism^88,89^. Oxytocin treatment in ASD patients increases NAc connectivity^90^ that was shown decreased notably as a function of OXTR risk-allele dosage:^19^ these data further highlight the connection between oxytocin activity and reward processing. Interestingly, our results bring additional arguments for combining pharmacological approaches targeting oxytocin with behavioral therapy^91,92^, by revealing shared neurobiological mechanisms.

Finally, behavioral intervention normalized (except in PFC) the expression of *Crh*, coding for corticotropin-releasing factor that possibly contributes to increased anxiety levels in ASD^93,94^, and partially restored striatal expression of *Grm4*, coding for the mGluR4 receptor, which activation rescues ASD symptoms in *Oprm1*^*−/−*^ mice^9^. These results indicate that the neurobiological underpinnings of therapy-induced behavioral improvements spread well over plasticity markers and the oxytocin/vasopressin system, and will require further investigation.

In conclusion, we demonstrate that reattributing a rewarding value to social experience in the *Oprm1*^*−/−*^ mouse model of ASD durably rescues deficient social abilities and modify neurobiological substrates in the reward circuitry, in accordance with the Social Motivation Theory of autism^95,96^. This occurred despite defective reward processing in mice lacking MOR, suggesting that palatable food pairing was able to rescue social reward in these animals, maybe by restoring oxytocin/vasopressin function. These results strongly suggest that the main key to success in EIBI programs is to restore social reward in patients with ASD. They also point to the therapeutic potential of pharmacological treatments stimulating reward processes to relieve ASD symptoms, an idea that is now emerging from clinical and imaging studies^21^. Such pharmacotherapy could target the oxytocin/vasopressin system as well as other key substrates for reward processing and efficiently complement EIBI programs by reducing their demand and the variability of their outcomes.

## Electronic supplementary material

Supplementary information

Table S1

Table S2

Table S3

Table S4

Table S5

Table S6

Table S7

Table S8

## References

[1] Le Merrer J, Becker JA, Befort K, Kieffer BL (2009). Reward processing by the opioid system in the brain. Physiol. Rev..

[2] Nogueiras R (2012). The opioid system and food intake: homeostatic and hedonic mechanisms. Obes. Facts.

[3] Panksepp J, Herman B, Conner R, Bishop P, Scott JP (1978). The biology of social attachments: opiates alleviate separation distress. Biol. Psychiatry.

[4] Smith CJW, Wilkins KB, Li S, Tulimieri MT, Veenema AH (2017). Nucleus accumbens mu opioid receptors regulate context-specific social preferences in the juvenile rat. Psychoneuroendocrinology.

[5] Burkett JP, Spiegel LL, Inoue K, Murphy AZ, Young LJ (2011). Activation of mu-opioid receptors in the dorsal striatum is necessary for adult social attachment in monogamous prairie voles. Neuropsychopharmacology.

[6] Loseth GE, Ellingsen DM, Leknes S (2014). State-dependent mu-opioid modulation of social motivation. Front. Behav. Neurosci..

[7] Pellissier LP, Gandia J, Laboute T, Becker JAJ, Le Merrer J (2018). Mu opioid receptor, social behaviour and autism spectrum disorder: reward matters. Br. J. Pharmacol.

[8] Vanderschuren LJ, Achterberg EJ, Trezza V (2016). The neurobiology of social play and its rewarding value in rats. Neurosci. Biobehav. Rev..

[9] Becker JA (2014). Autistic-like syndrome in mu opioid receptor null mice is relieved by facilitated mGluR4 activity. Neuropsychopharmacology.

[10] Cinque C (2012). Modeling socially anhedonic syndromes: genetic and pharmacological manipulation of opioid neurotransmission in mice. Transl. Psychiatry.

[11] Gigliucci V (2014). Region specific up-regulation of oxytocin receptors in the opioidoprm1 (−/−) mouse model of autism. Front. Pediatr..

[12] Moles A, Kieffer BL, D’Amato FR (2004). Deficit in attachment behavior in mice lacking the mu-opioid receptor gene. Science.

[13] Oddi D, Crusio WE, D’Amato FR, Pietropaolo S (2013). Monogenic mouse models of social dysfunction: implications for autism. Behav. Brain. Res..

[14] Lai MC, Lombardo MV, Baron-Cohen S (2014). Autism. Lancet.

[15] American Psychiatric Association. *Diagnostic and statistical manual of mental disorders*, 5th edn. Arlington, VA : American Psychiatric Publishing, 2013.

[16] Argyropoulos A, Gilby KL, Hill-Yardin EL (2013). Studying autism in rodent models: reconciling endophenotypes with comorbidities. Front. Hum. Neurosci..

[17] Mechling AE (2016). Deletion of the mu opioid receptor gene in mice reshapes the reward-aversion connectome. Proc. Natl Acad. Sci. USA.

[18] Dichter GS (2012). Reward circuitry function in autism spectrum disorders. Soc. Cogn. Affect. Neurosci..

[19] Hernandez LM (2017). Additive effects of oxytocin receptor gene polymorphisms on reward circuitry in youth with autism. Mol. Psychiatry.

[20] Kohls G, Yerys BE, Schultz RT (2014). Striatal development in autism: repetitive behaviors and the reward circuitry. Biol. Psychiatry.

[21] Kohls G, Antezana L, Mosner MG, Schultz RT, Yerys BE (2018). Altered reward system reactivity for personalized circumscribed interests in autism. Mol. Autism.

[22] Chevallier C, Grezes J, Molesworth C, Berthoz S, Happe F (2012). Brief report: Selective social anhedonia in high functioning autism. J. Autism Dev. Disord..

[23] Dawson, G. & Bernier, R. in *Human behavior and the developing brain: Atypical development*. 2nd edn (eds D. Coch, G. Dawson, K. Fischer) 28–55 (Guilford Press, New York, 2007).

[24] Dove D (2012). Medications for adolescents and young adults with autism spectrum disorders: a systematic review. Pediatrics.

[25] Ji N, Findling RL (2015). An update on pharmacotherapy for autism spectrum disorder in children and adolescents. Curr. Opin. Psychiatry.

[26] Warren Z (2011). A systematic review of early intensive intervention for autism spectrum disorders. Pediatrics.

[27] Allen-Meares P, MacDonald M, McGee K (2016). Autism spectrum disorder updates - relevant information for early interventionists to consider. Front. Public Health.

[28] Reichow B (2012). Overview of meta-analyses on early intensive behavioral intervention for young children with autism spectrum disorders. J. Autism Dev. Disord..

[29] Pickles A (2016). Parent-mediated social communication therapy for young children with autism (PACT): long-term follow-up of a randomised controlled trial. Lancet.

[30] Weston R, Hodges A, Davis TN (2017). Differential reinforcement of other behaviors to treat challenging behaviors among children with autism: a systematic and quality review. Behav. Modif..

[31] Matson JL, Boisjoli JA (2009). The token economy for children with intellectual disability and/or autism: a review. Res. Dev. Disabil..

[32] Lovaas OI (1987). Behavioral treatment and normal educational and intellectual functioning in young autistic children. J. Consult. Clin. Psychol..

[33] Koegel LK, Koegel RL, Carter CM (1998). Pivotal responses and the natural language teaching paradigm. Semin. Speech Lang..

[34] Jensen VK, Sinclair LV (2002). Treatment of autism in young children: behavioral intervention and applied behavior analysis. Infant Young Child..

[35] Bradshaw J, Steiner AM, Gengoux G, Koegel LK (2015). Feasibility and effectiveness of very early intervention for infants at-risk for autism spectrum disorder: a systematic review. J. Autism Dev. Disord..

[36] Dawson G (2010). Randomized, controlled trial of an intervention for toddlers with autism: the Early Start Denver model. Pediatrics.

[37] Kaiser AP, Hester PP (1994). Generalized effects of enhanced Milieu teaching. J. Speech Hear. Res..

[38] Koegel RL, Koegel LK, McNerney EK (2001). Pivotal areas in intervention for autism. J. Clin. Child Psychol..

[39] Wetherby AM (2014). Parent-implemented social intervention for toddlers with autism: an RCT. Pediatrics.

[40] Ilg J (2018). Evaluation of a French perent-training program in young children with autism spectrum disorder. Psychologie Française.

[41] Lei J, Ventola P (2017). Pivotal response treatment for autism spectrum disorder: current perspectives. Neuropsychiatr. Dis. Treat..

[42] Johnson KA, Vladescu JC, Kodak T, Sidener TM (2017). An assessment of differential reinforcement procedures for learners with autism spectrum disorder. J. Appl. Behav. Anal..

[43] Leaf JB (2016). Changing preference from tangible to social activities through an observation procedure. J. Appl. Behav. Anal..

[44] Smith T, Klorman R, Mruzek DW (2015). Predicting outcome of community-based early intensive behavioral intervention for children with autism. J. Abnorm. Child Psychol..

[45] Estes A (2015). Long-term outcomes of early intervention in 6-year-old children with autism spectrum disorder. J. Am. Acad. Child Adolesc. Psychiatry.

[46] Linstead E (2017). An evaluation of the effects of intensity and duration on outcomes across treatment domains for children with autism spectrum disorder. Transl. Psychiatry.

[47] Matthes HW (1996). Loss of morphine-induced analgesia, reward effect and withdrawal symptoms in mice lacking the mu-opioid-receptor gene. Nature.

[48] Fisher W (1992). A comparison of two approaches for identifying reinforcers for persons with severe and profound disabilities. J. Appl. Behav. Anal..

[49] Crawley JN (2007). Mouse behavioral assays relevant to the symptoms of autism. Brain. Pathol..

[50] Silverman JL, Yang M, Lord C, Crawley JN (2010). Behavioural phenotyping assays for mouse models of autism. Nat. Rev. Neurosci..

[51] Moustgaard A, Hau J, Lind NM (2008). Effects of dopamine D4 receptor antagonist on spontaneous alternation in rats. Behav. Brain Funct..

[52] Thomas A (2009). Marble burying reflects a repetitive and perseverative behavior more than novelty-induced anxiety. Psychopharmacol. (Berl.).

[53] Meirsman AC (2016). Mice lacking GPR88 show motor deficit, improved spatial learning and low anxiety reversed by delta opioid antagonist. Biol. Psychiatry.

[54] Becker JA, Kieffer BL, Le Merrer J (2017). Differential behavioral and molecular alterations upon protracted abstinence from cocaine versus morphine, nicotine, THC and alcohol. Addict. Biol..

[55] Ventola P (2015). Heterogeneity of neural mechanisms of response to pivotal response treatment. Brain. Imaging Behav..

[56] Yang D (2016). Brain responses to biological motion predict treatment outcome in young children with autism. Transl. Psychiatry.

[57] Papaleo F, Kieffer BL, Tabarin A, Contarino A (2007). Decreased motivation to eat in mu-opioid receptor-deficient mice. Eur. J. Neurosci..

[58] Pecina S, Berridge KC (2005). Hedonic hot spot in nucleus accumbens shell: where do mu-opioids cause increased hedonic impact of sweetness?. J. Neurosci..

[59] Pecina S, Berridge KC (2000). Opioid site in nucleus accumbens shell mediates eating and hedonic ‘liking’ for food: map based on microinjection Fos plumes. Brain Res..

[60] Olmstead MC, Ouagazzal AM, Kieffer BL (2009). Mu and delta opioid receptors oppositely regulate motor impulsivity in the signaled nose poke task. PLoS. ONE.

[61] Karsten AM, Carr JE (2009). The effects of differential reinforcement of unprompted responding on the skill acquisition of children with autism. J. Appl. Behav. Anal..

[62] Fisher WW, Pawich TL, Dickes N, Paden AR, Toussaint K (2014). Increasing the saliency of behavior-consequence relations for children with autism who exhibit persistent errors. J. Appl. Behav. Anal..

[63] Kim SH, Bal VH, Lord C (2017). Longitudinal follow-up of academic achievement in children with autism from age 2 to 18. J. Child Psychol. Psychiatry.

[64] Garbugino L, Centofante E, D’Amato FR (2016). Early social enrichment improves social motivation and skills in a monogenic mouse model of autism, the Oprm1 (−/−) mouse. Neural Plast..

[65] Pellecchia M (2016). Child characteristics associated with outcome for children with autism in a school-based behavioral intervention. Autism.

[66] Trezza V, Baarendse PJ, Vanderschuren LJ (2010). The pleasures of play: pharmacological insights into social reward mechanisms. Trends Pharmacol. Sci..

[67] Kohls G, Chevallier C, Troiani V, Schultz RT (2012). Social ‘wanting’ dysfunction in autism: neurobiological underpinnings and treatment implications. J. Neurodev. Disord..

[68] Dichter GS, Damiano CA, Allen JA (2012). Reward circuitry dysfunction in psychiatric and neurodevelopmental disorders and genetic syndromes: animal models and clinical findings. J. Neurodev. Disord..

[69] Dalton KM (2005). Gaze fixation and the neural circuitry of face processing in autism. Nat. Neurosci..

[70] Li Y (2015). A critical evaluation of the activity-regulated cytoskeleton-associated protein (Arc/Arg3.1)‘s putative role in regulating dendritic plasticity, cognitive processes, and mood in animal models of depression. Front. Neurosci..

[71] Plath N (2006). Arc/Arg3.1 is essential for the consolidation of synaptic plasticity and memories. Neuron.

[72] Kang MS (2017). Autism-like behavior caused by deletion of vaccinia-related kinase 3 is improved by TrkB stimulation. J. Exp. Med..

[73] Cao C (2013). Impairment of TrkB-PSD-95 signaling in Angelman syndrome. PLoS Biol..

[74] Alhowikan AM (2016). Activity-regulated cytoskeleton-associated protein dysfunction may contribute to memory disorder and earlier detection of autism spectrum disorders. Med. Princ. Pract..

[75] Manago F (2016). Genetic disruption of Arc/Arg3.1 in mice causes alterations in dopamine and neurobehavioral phenotypes related to schizophrenia. Cell Rep..

[76] Huentelman MJ (2015). Association of SNPs in EGR3 and ARC with schizophrenia supports a biological pathway for schizophrenia risk. PLoS. ONE.

[77] Purcell SM (2014). A polygenic burden of rare disruptive mutations in schizophrenia. Nature.

[78] Fromer M (2014). De novo mutations in schizophrenia implicate synaptic networks. Nature.

[79] Manago F, Papaleo F (2017). Schizophrenia: what’s Arc got to do with It?. Front. Behav. Neurosci..

[80] Calderoni S (2016). Rehabilitative interventions and brain plasticity in autism spectrum disorders: focus on mri-based studies. Front. Neurosci..

[81] Knobloch HS, Grinevich V (2014). Evolution of oxytocin pathways in the brain of vertebrates. Front. Behav. Neurosci..

[82] Bigler RL, Kamande JW, Dumitru R, Niedringhaus M, Taylor AM (2017). Messenger RNAs localized to distal projections of human stem cell derived neurons. Sci. Rep..

[83] Meyer-Lindenberg A, Domes G, Kirsch P, Heinrichs M (2011). Oxytocin and vasopressin in the human brain: social neuropeptides for translational medicine. Nat. Rev. Neurosci..

[84] Johnson ZV, Young LJ (2017). Oxytocin and vasopressin neural networks: Implications for social behavioral diversity and translational neuroscience. Neurosci. Biobehav. Rev..

[85] Penagarikano O (2017). Oxytocin in animal models of autism spectrum disorder. Dev. Neurobiol..

[86] Sala M (2011). Pharmacologic rescue of impaired cognitive flexibility, social deficits, increased aggression, and seizure susceptibility in oxytocin receptor null mice: a neurobehavioral model of autism. Biol. Psychiatry.

[87] Kosaki Y, Watanabe S (2016). Conditioned social preference, but not place preference, produced by intranasal oxytocin in female mice. Behav. Neurosci..

[88] Guastella AJ, Hickie IB (2016). Oxytocin treatment, circuitry, and autism: a critical review of the literature placing oxytocin into the autism context. Biol. Psychiatry.

[89] Umbricht D (2017). A single dose, randomized, controlled proof-of-mechanism study of a novel vasopressin 1a receptor antagonist (RG7713) in high-functioning adults with autism spectrum disorder. Neuropsychopharmacology.

[90] Gordon I (2016). Intranasal oxytocin enhances connectivity in the neural circuitry supporting social motivation and social perception in children with autism. Sci. Rep..

[91] Stavropoulos KK, Carver LJ (2013). Research review: Social motivation and oxytocin in autism--implications for joint attention development and intervention. J. Child Psychol. Psychiatry.

[92] Hall SS, Lightbody AA, McCarthy BE, Parker KJ, Reiss AL (2012). Effects of intranasal oxytocin on social anxiety in males with fragile X syndrome. Psychoneuroendocrinology.

[93] McGill BE (2006). Enhanced anxiety and stress-induced corticosterone release are associated with increased Crh expression in a mouse model of Rett syndrome. Proc. Natl Acad. Sci. USA.

[94] Tsilioni I (2014). Elevated serum neurotensin and CRH levels in children with autistic spectrum disorders and tail-chasing Bull Terriers with a phenotype similar to autism. Transl. Psychiatry.

[95] Chevallier C, Kohls G, Troiani V, Brodkin ES, Schultz RT (2012). The social motivation theory of autism. Trends Cogn. Sci..

[96] Dawson G (2008). Early behavioral intervention, brain plasticity, and the prevention of autism spectrum disorder. Dev. Psychopathol..

