# Supplementary material for: Back-translating behavioral intervention for autism spectrum disorders to mice with blunted reward restores social abilities
Source: Transl Psychiatry. 2018 Sep 21;8:197. doi: 10.1038/s41398-018-0247-y (PMC6155047; doi:10.1038/s41398-018-0247-y)
Supplement: Supplementary file 1 — Supplementary information [file 41398_2018_247_MOESM1_ESM.docx]

**Back-translating behavioral intervention for autism spectrum disorders to mice with blunted reward restores social abilities**

Camille Pujol^†^, Lucie P. Pelissier^†^, Céline Clément, Jérôme Becker*, Julie Le Merrer*

**Supplementary experimental procedures**

**Behavioral experiments**

*Behavioral training protocol*

Equivalent numbers of naïve male and female animals were used in each experimental group. Female mice were not synchronized for estrous cycle. Experiments started when mice were 6-week old to mimic conditions of an early behavioral intervention. Caregivers in EIBI programs initially define several target behaviors to work on; here we focused on social interaction. Animals were randomly distributed across four experimental conditions before behavioral assays had started: reinforced object interaction (R-OI), non-reinforced social interaction (NR-SI) and reinforced social interaction (R-SI) (see details below and in Figure 1a). Behavioral testing was performed in three steps (time line in Figure 1b).

*Pre-training tests:* Before starting behavioral intervention, we assessed the preference of the animals for a food reinforcer and evaluated perseverative behavior and social abilities using the Y-maze exploration and direct social interaction tests (post-natal - PN - week 6).

First, we prepared two different highly palatable food reinforcers by boiling condensed milk or peanut butter with 5% alimentary agar-agar powder. The solidified preparations obtained were then portioned in ~3-g units and both presented in the home cage of the animals in their fifth week of age for 5 consecutive days to allow habituation. On days 1 and 2 of PN week 6, the mice were introduced once daily in a Y-maze for 5 min of free exploration. On day 3, a 3-g unit of each food reinforcer was randomly placed in two arms of the maze, the third arm remaining empty. The animals were allowed to explore the maze for 5 min. The time spent in each arm was scored on video recordings and used to assess preference for the reinforcers. The preferred reinforcer was then used for all consecutive experiments in experimental groups receiving a food reinforcer (R-OI and R-SI).

Perseverative behavior was assessed by measuring spontaneous alternation on video recordings from the first session of habituation in the Y-maze (PN week 6, day 1). A direct social interaction test was performed on day 5, after habituation to the arenas on day 4 (30 min). Each experimental animal then met an aged-, sex- and genotype-matched non cage mate animal from the same experimental group (see below for detailed testing protocols).

*Behavioral training:* Behavioral intervention started on week 7 and lasted 3 weeks. Experiments were performed in the same arenas and under the same lighting conditions as the pre-intervention social interaction test. In a first group, the SI-R condition, mutant and wild-type mice interacted 5 days a week with a wild-type unfamiliar conspecific, different every day, for 5 min during the two first weeks, for 8 min during the third week. The interactor was then removed from the arena while the experimental animal stayed there as long as the interaction lasted (5 or 8 min) and received a ~3 g unit of its favorite food reward. The amount of food was weighted before and after the test to measure consumption. In a first control group, the SI-NR condition, intervention was performed in the exact same conditions except that no food reward was available in the arenas following social interaction. In SI-NR and SI-R groups, the time spent in nose contacts and their duration were measured during the course of intervention to assess the evolution of social behavior (days 2,4,8,12 and 14 of the intervention). During the two last training sessions, some male mice (*Oprm1^+/+^* and *Oprm1^-/-^*) from the SI-R group developed aggressive behavior, maybe a territorial response due to repetitive food presentation in the arena. In a second control group, the OI-R condition, food reward was offered to the animals after exposure to an unfamiliar object (different every day) instead of a mouse. The amount of food consumed was measured. Finally, in a no therapy control group (control condition), the animals were tested on weeks 6, 10 and 11 under the same conditions as in the other groups, but were not manipulated during weeks 7-9.

*Post-training tests:* Beginning on week 10, we performed a battery of behavioral assays over two weeks to assess the consequences of intervention. Social abilities were explored using the direct social interaction (between unfamiliar animals of the same genotype and experimental group) and three-chamber tests[^1^](#_ENREF_1). Stereotyped/perseverative behavior was assessed by scoring motor stereotypies[^2^](#_ENREF_2), monitoring alternation in a Y-maze[^3^](#_ENREF_3) and assessing anxiety-induced marble burying[^4^](#_ENREF_4). Anxiety was evaluated in the novelty-suppressed feeding test[^5^](#_ENREF_5) (testing order in Figure S1). Detailed behavioral protocols for each test are described below.

In cohorts dedicated to qRT-PCR analysis (half of the OI-R, SI-NR and SI-R cohorts), animals were submitted to 3 additional days of behavioral intervention (week 12) during which they meet with gender-matched wild-type conspecifics, and sacrificed 45 min after the beginning of a last social interaction test (5 min), without food presentation (Figure 1b) during which they met gender and genotype-matched animals. To assess the maintenance of intervention effects over the time, we submitted half of the SI-NR and SI-R cohorts to a direct social interaction test on weeks 17 and 24 (detailed protocol below). During these tests, each animal met with a gender and genotype-matched mouse.

**Behavioral testing**

Direct social interaction and novelty suppressed feeding were performed in 4 equal square arenas (50 x 50 cm) separated by 35cm-high opaque grey Plexiglas walls over a white Plexiglas platform (infrared floor, View Point, Lyon, France).

***Social abilities***

**Direct social interaction test.** On testing day, a pair of unfamiliar mice (not cage mates) was introduced in each arena for 10 min (15 lx). Each arena received a black plastic floor (transparent to infrared) covered with lightly sprayed fresh sawdust to limit anxiety. The total amount of time spent in nose contact (nose-to-nose, nose-to-body or nose-to-anogenital region), the number and duration of these contacts, grooming episodes (allogrooming), notably ones occurring immediately (<5s) after a social contact, as well as the number of following episodes were scored *a posteriori* on video recordings (infrared light-sensitive video camera) [^6-8^](#_ENREF_6) using an ethological keyboard (Labwatcher®, View Point, Lyon, France) by trained experimenters and individually for each animal. The mean duration of nose contacts was calculated from previous data [^9-11^](#_ENREF_9).

During pre- and post-intervention tests, each animal met an age-, sex- and genotype-matched non cage mate animal from the same experimental group (control, OI-R, SI-NR or SI-R). During behavioral intervention, each animal, *Oprm1^+/+^* or *Oprm1^-/-^*, met a wild-type age- and sex-matched unfamiliar conspecific (from the same mouse line), different every day.

**Three-chamber social preference test.** The test apparatus consisted of a transparent acrylic box (exterior walls blinded with black plastic film); partitions divided the box into three equal chambers (40 x 20 x 22.5 cm). Two sliding doors (8 x 5 cm) allowed transitions between chambers. Cylindrical wire cages (18 x 9 cm, 0.5 cm diameter-rods spaced 1 cm apart) were used to contain the mouse interactor and object (soft-toy mouse). The test was performed in low-light conditions (15 lx) to minor anxiety. Stimulus wild-type mice were habituated to confinement in wire cages for 2 days before the test (20 min/day). On testing day, the experimental animal was introduced to the middle chamber and allowed to explore the whole apparatus for a 10-min habituation phase (wire cages empty) after the sliding doors were raised. The experimental mouse was then confined back in the middle-chamber while the experimenter introduced an unfamiliar wild type gender-matched animal into a wire cage in one of the side-chambers and a soft toy mouse (8 x 10 cm) in the second wire cage as a control for novelty. Then the experimental mouse was allowed to explore the apparatus for a 10-min interaction phase. The time spent in each chamber, the time spent in close contact (nose or paw contact) with each wire cage (empty: habituation; containing a mouse or a toy: interaction), as well as the number of these close contacts was scored *a posteriori* on video recordings using an ethological keyboard (Labwatcher®, View Point, Lyon, France) by trained experimenters. The mean duration of close contacts was calculated from these data [^9-11^](#_ENREF_9). The relative position of stimulus mice (versus toy) was counterbalanced between groups [^6^](#_ENREF_6)^,^ [^12^](#_ENREF_12). Animals that failed to enter both lateral chambers of the three-chamber apparatus during the exploration phase were excluded from the analysis (2 mice of the NoT group were excluded: one male *Oprm1^+/+^* and one male *Oprm1^-/-^*).

***Stereotyped behaviors***

**Motor stereotypies.** To detect motor stereotypies in animals from the different experimental groups, we placed mice individually in clear standard home cages (21×11×17 cm) covered with a stainless steel grid and filled with 3-cm deep fresh sawdust for 10 min [^2^](#_ENREF_2). Light intensity was set at 30 lux. The number of head shakes and rearing, burying, grooming, circling episodes as well as the total amount of time spent burying were scored by direct observation. This scoring was only performed by properly trained experimenters.

**Y-maze exploration.** Spontaneous alternation behaviour was used to assess perseverative behavior[^3^](#_ENREF_3)^,^ [^13^](#_ENREF_13)^,^ [^14^](#_ENREF_14). Each Y-maze consisted of three connected Plexiglas arms (15x15x17 cm) covered with distinct wall patterns (15 lx). Floors were covered with lightly sprayed fresh sawdust to limit anxiety. Each mouse was placed at the center of a maze and allowed to freely explore this environment for 6 min. The pattern of entries into each arm was quoted on video-recordings. Spontaneous alternations (SPA), i.e. successive entries into each arm forming overlapping triplet sets, alternate arm returns (AAR) and same arm returns (SAR) were scored, and the percentage of SPA, AAR and SAR was calculated as following: total / (total arm entries -2) * 100 [^5^](#_ENREF_5)^,^ [^6^](#_ENREF_6).

**Marble-burying.** Marble burying was used as a measure of perseverative behavior[^4^](#_ENREF_4). Mice were introduced individually in transparent cages (21×11×17 cm) containing 20 glass marbles (diameter: 1.5 cm) evenly spaced on 4-cm deep fresh sawdust. To prevent escapes, each cage was covered with a filtering lid. Light intensity in the room was set at 40 lux. The animals were removed from the cages after 15 min, and the number of marbles buried more than half in sawdust was quoted [^5^](#_ENREF_5)^,^ [^6^](#_ENREF_6).

***Anxiety-like behavior***

**Novelty-suppressed feeding.** Novelty-suppressed feeding (NSF) was measured in 24-hr food-deprived mice, isolated in a standard housing cage for 30 min before individual testing. Three pellets of ordinary lab chow were placed on a white tissue in the center of each arena, lit at 60 lx. Each mouse was placed in a corner of an arena and allowed to explore for a maximum of 15 min. Latency to feed was measured as the time necessary to bite a food pellet. Immediately after an eating event, the mouse was transferred back to home cage (free from cage-mates) and allowed to feed on lab chow for 5 min. Food consumption in the home cage was measured [^5^](#_ENREF_5)^,^ [^6^](#_ENREF_6)^,^ [^15^](#_ENREF_15).

**Supplementary Figures**


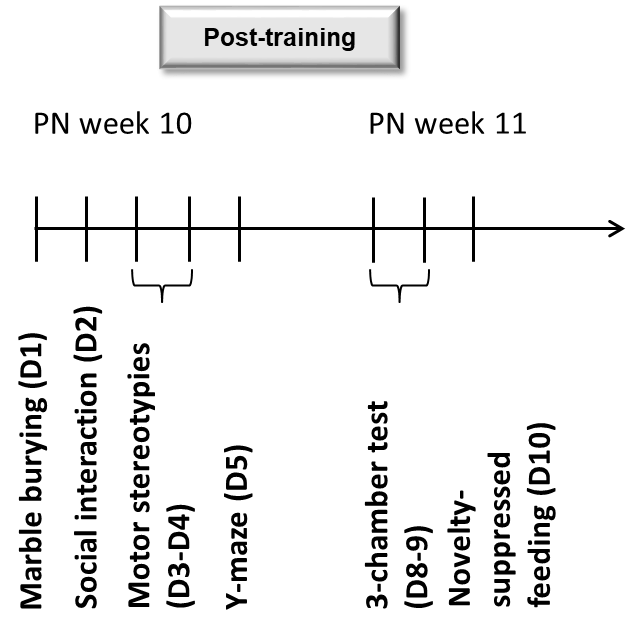


**Figure S1. Testing order during post-training assays.** Testing order was identical between the different cohorts and chosen to limit the impact of stress on further behavioral assessments. D: day; PN: post-natal.


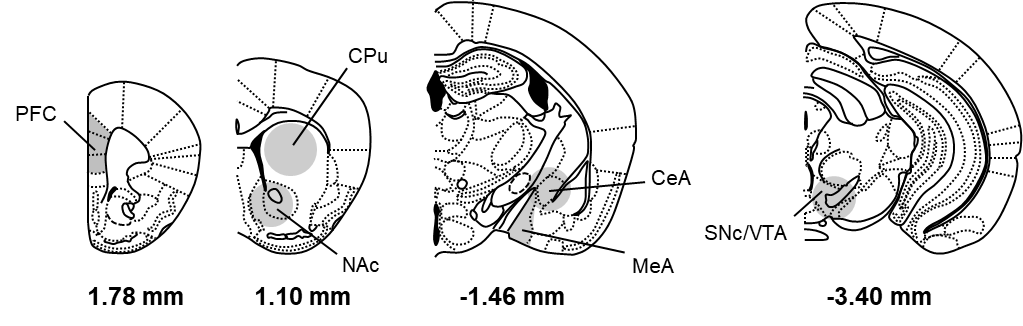


**Figure S2. Schematic representations depict brain regions dissected for gene expression study.** PFC, CPu, NAc, CeA, and VTA/SNc were punched on 1-mm thick brain slices (PFC: one bilateral punch, ᴓ 2 mm; CPu, NAc, CeA, and VTA/SNc: one punch/side, ᴓ 1.2 mm). MeA was dissected out on brain slices. Coordinates refer to bregma. CPu: caudate putamen; CeA: central amygdala; NAc: nucleus accumbens; PFC: prefrontal cortex; SNc: substancia nigra, pars compacta; VTA: ventral tegmental area.


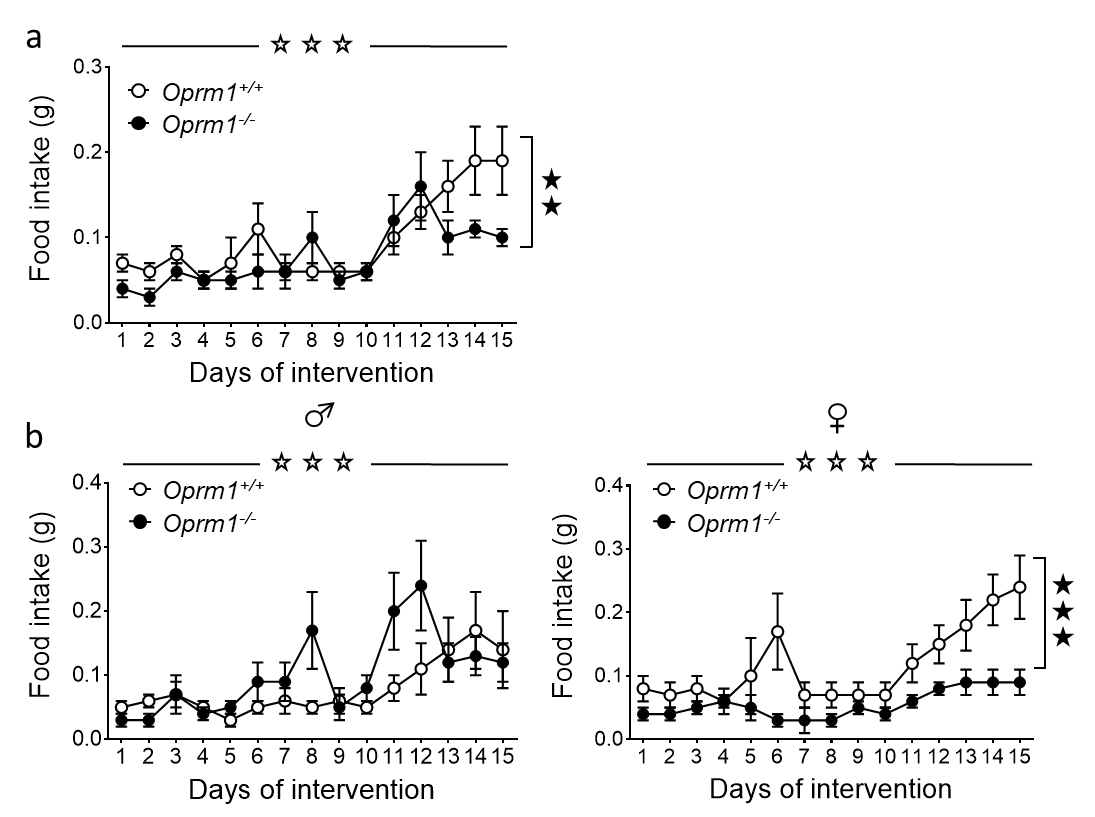


**Figure S3. Palatable food intake increased along the course of behavioral training in *Oprm1^+/+^* and *Oprm1^-/-^* mice.** (a) Over the days of intervention, *Oprm1^+/+^* (open dots) and *Oprm1^-/-^* (solid dots) animals increased their food intake, with mutant mice consuming less of their favorite food reward than their wild-type counterparts did. (b) Differences between genotypes in food consumption during training was influenced by sex, with mutant female mice consuming significantly less food than female wild-type animals, whereas male *Oprm1^-/-^* ate similar amounts of palatable food than male *Oprm1^+/+^* controls. Animal numbers per genotype and gender: OI-R condition, n=8; SI-R condition, n=10-11. Open stars: training effect; solid stars: genotype effect (four-way ANOVA with days of intervention as a repeated measure: training effect); one star: p<0.05, two stars: p<0.01, three stars: p<0.001. See Table S4 for statistics.

**
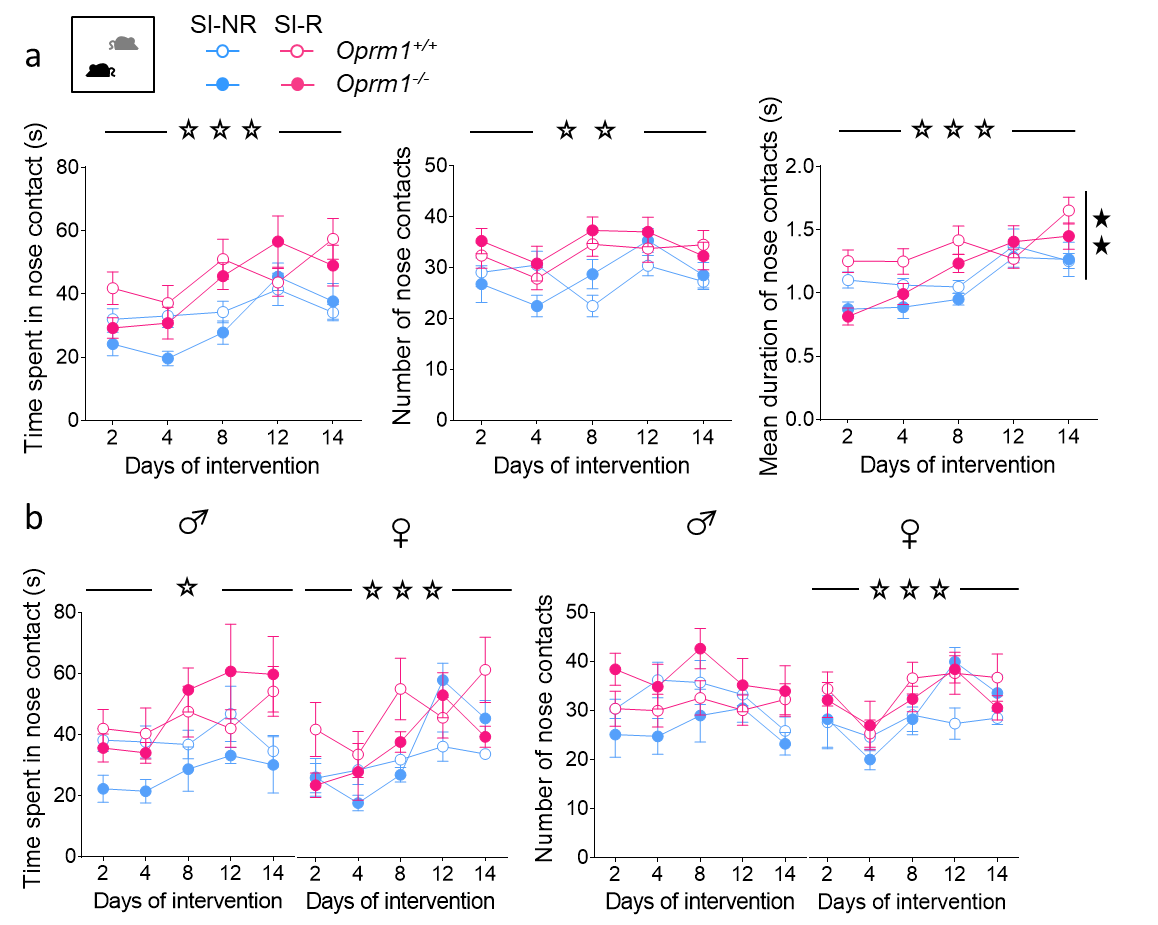
**

**Figure S4. Social interaction parameters were modified along the course of behavioral training in *Oprm1^+/+^* and *Oprm1^-/-^* mice.** (a) During social interaction sessions, the number of and time spent in nose contact(s) (NC) increased over time in wild-type and mutant mice from both SI-NR and SI-R groups; the mean duration of NC in the SI-R group was higher than in the SI-NR group. (b) The effects of training on the time spent in NC was influenced by sex, with female mutant mice spending more time in NC under the SI-NR condition than males. Number of NC over training was significantly increased in female but not male mice. Animal numbers per genotype and gender: SI-NR condition, n=8; SI-R condition, n=10-11. Data are presented as mean ± SEM. Open stars: training effect; solid stars: genotype effect (four-way ANOVA with days of intervention as a repeated measure: training effect); one star: p<0.05, two stars: p<0.01, three stars: p<0.001. SI-NR: Social interaction – non reinforced; SI-R: social interaction – reinforced. See Table S4 for statistics.

**
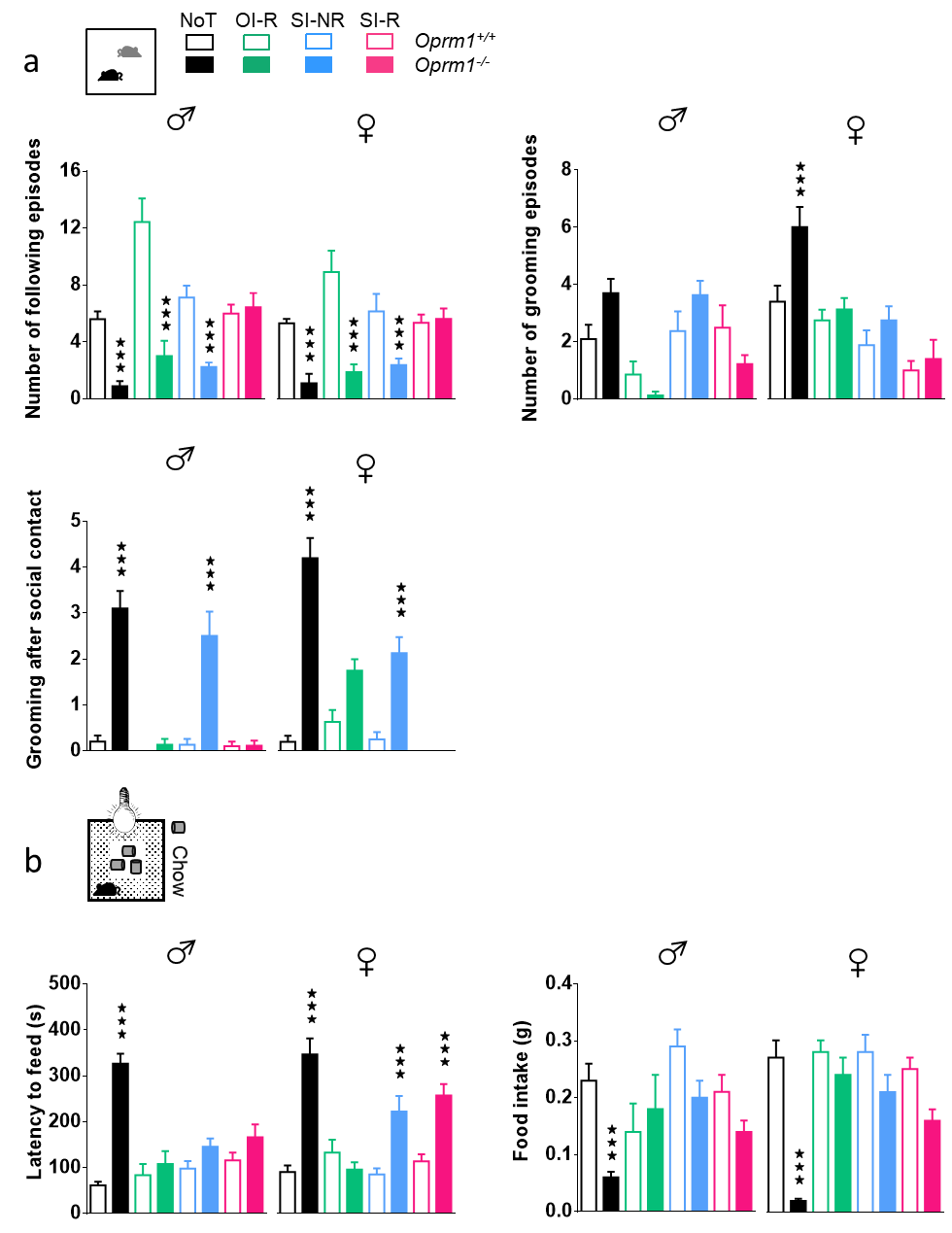
**

**Figure S5. Influence of gender on social interaction and novelty-suppressed feeding parameters following behavioral training.** (a) When tested for direct social interaction after behavioral training, male *Oprm1^+/+^* mice from the OI-R group displayed more following episodes than female *Oprm1^+/+^* mice in the same group. As regards grooming episodes, female *Oprm1^-/-^* mice groomed more often than their wild-type counterparts, and globally female mice trained under the NoT and OI-R groomed more than males during the test. Similarly, female mutant mice from the NoT group groomed more often after a social contact than their male equivalent, and both wild-type and knockout female animals trained under the OI-R condition groomed more than their male equivalents. However, in this test, both male and female *Oprm1^-/-^* mouse populations similarly beneficiated from behavioral intervention (SI-R condition: increased number of followings and reduced grooming episodes, notably after a social contact). (b) When tested for novelty-suppressed feeding after behavioral training, male *Oprm1^-/-^* mice trained under the OI-R, SI-NR and SI-R conditions displayed reduced latencies to eat as compared to the same animals trained under the NoT condition. In contrast, female mutant animals only displayed normalized feeding latencies when trained under the OI-R condition. Thus repeated handling and training in male *Oprm1^-/-^* mice was sufficient to reduced anxiety in this test whereas only training under the OI-R condition allowed such reduction in females. As regards food intake, female mice ate more than males when returned to their home cage. Animal numbers per genotype and gender: NoT condition, n=9-10; OI-R condition, n=7-8; SI-NR condition, n=8; SI-R condition, n=10-11. Data are presented as mean ± SEM. Solid stars: genotype x condition interaction (three-way ANOVA followed by Newman-Keules post-hoc test); one star: p<0.05, two stars: p<0.01, three stars: p<0.001. SI-NR: Social interaction – non reinforced; SI-R: social interaction – reinforced. See Table S5 for statistics.

**
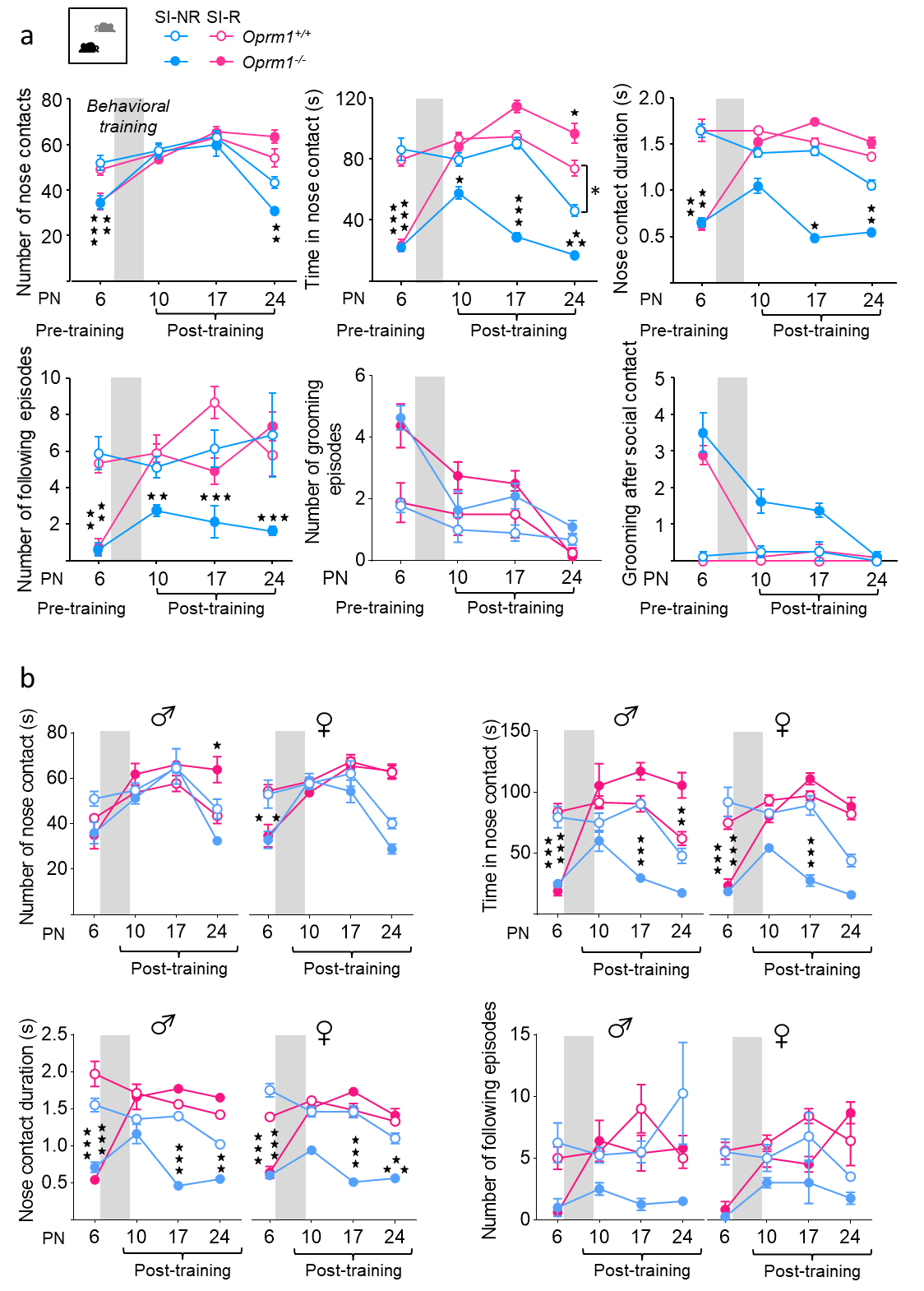
**

**Figure S6. Behavioral intervention (SI-R) durably relieved social interaction deficit in male and female *Oprm1^-/-^* mice.** (a) Beneficial effects of behavioral training on social interaction parameters in mutant animals were maintained up to 24 weeks after complete cessation of behavioral training in knockout mice from the SI-R and not SI-NR group. *Oprm1^+/+^* animals trained under the SI-R condition also maintained greater levels of social interaction over time than their SI-NR counterparts did. (b) Several social interaction parameters along repeated testing were influenced by sex. Beneficial effects of behavioral intervention (SI-R) and their maintenance over time, however, did not significantly differ between male and female mutant animals. Data are presented as mean ± SEM. Animal numbers per genotype and gender: SI-NR condition: n=4; SI-R condition: n=4-6. Genotype x condition interaction, solid stars: compared to wild-type animals trained under the same condition; asterisk: *Oprm1^+/+^* SI-R compared to *Oprm1^+/+^* SI-NR (four-way ANOVA with week of testing as a repeated measure, followed by Newman-Keules post-hoc test). One symbol: p<0.05, two symbols: p<0.01, three symbols: p<0.001. NoT: no therapy; SI-NR: Social interaction – non reinforced; SI-R: social interaction – reinforced.


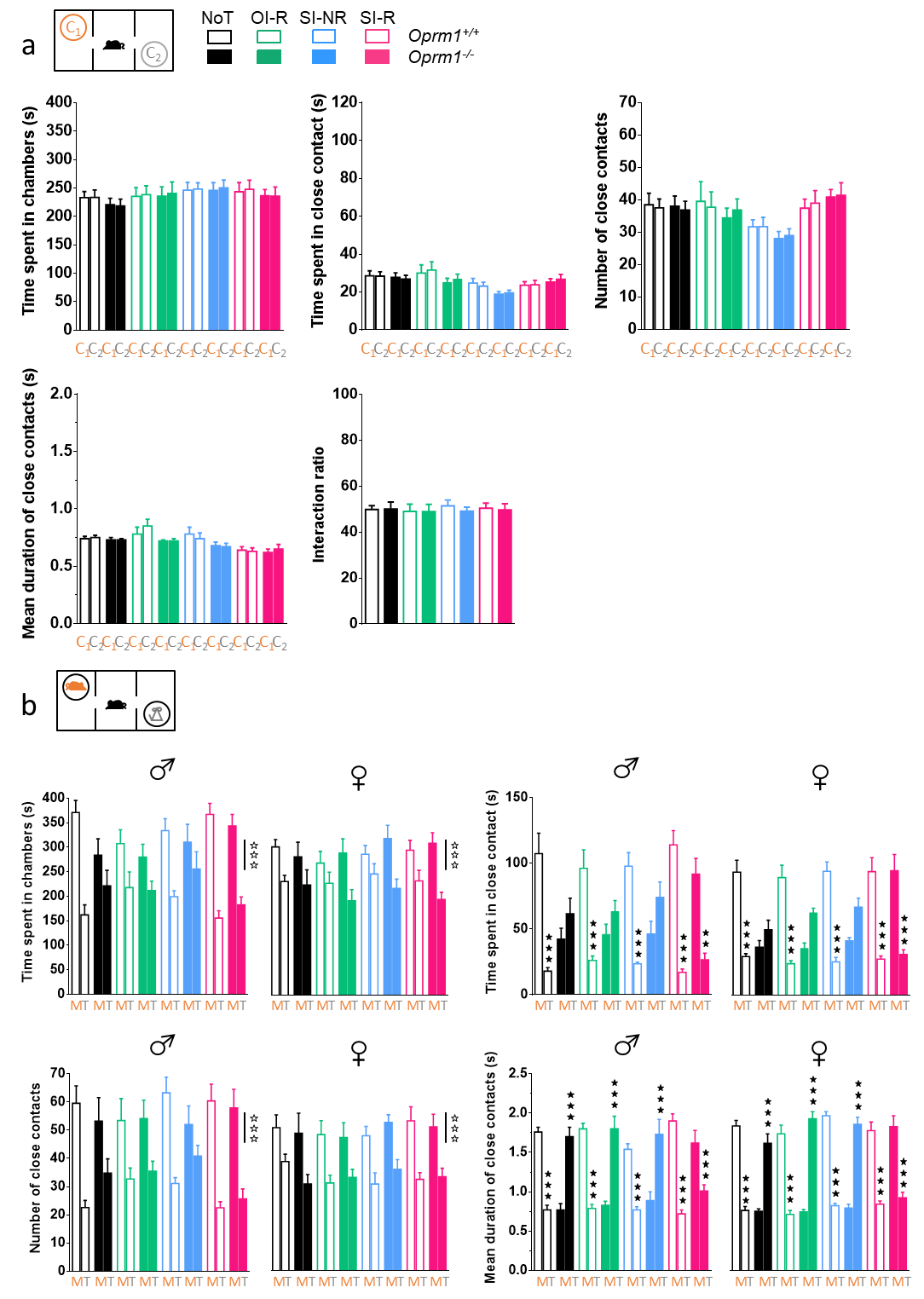


**Figure S7. Preference parameters measured during the habituation of the 3-chamber test and sex effects detected during the interaction phase.** (a) *Oprm1^+/+^* and *Oprm1^-/-^* mice showed no preference in spending time in one of the chambers over the other, or in exploring one wired cage over the other, whatever the training condition was. Two mice (NoT condition) failed to explore both chambers of the 3-chamber apparatus during the habituation phase and were excluded from the analysis. (b) Gender had a significant influence on several of the parameters measured during the interaction phase of the 3-chamber test. Globally, the difference in exploring the chamber with the mouse or the mouse itself versus the chamber with the toy or the toy itself (time in chambers, time spent in close contact, number of contacts and mean duration of contacts) was more pronounced in male than female mice, especially in wild-type animals. However, beneficial effects of behavioral intervention (SI-R) were similarly detectable in both male and female mutant animals. Data are presented as mean ± SEM. Animal numbers per genotype and gender: NoT condition, n=9-10; OI-R condition, n=7-8; SI-NR condition, n=8; SI-R condition, n=10-11. Solid stars: genotype x condition interaction, comparison to wild-type animals treated under the same condition; open stars: genotype effect (four-way ANOVA with one repeated measure: stimulus - mouse versus toy followed by Newman-Keules post-hoc test). Three stars: p<0.001. C1: cage 1; C2: cage 2; M: mouse; T: toy. NoT: no therapy; OI-R: object interaction – reinforced; SI-NR: Social interaction – non reinforced; SI-R: social interaction – reinforced. See Table S5 for statistics.


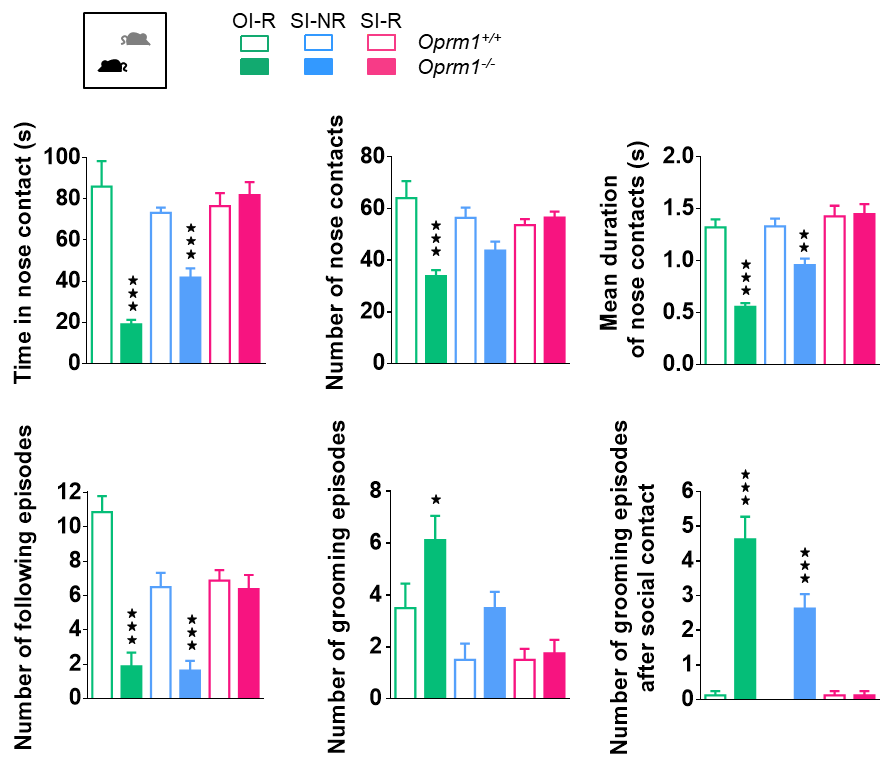


**Figure S8. Social interaction parameters measured 45 min before qRT-PCR experiments.** In cohorts dedicated to qRT-PCR analysis (half of the OI-R, SI-NR and SI-R cohorts), animals were submitted to three additional days of behavioral training after post-training tests (Figure 1b), and sacrificed 45 min after the beginning of an additional social interaction session without food presentation. As seen immediately after training (Figure 3a), time spent in NC, number of NC and mean duration of NC were partially or completely restored to wild-type levels in *Oprm1^-/-^* mice from the SI-NR and SI-R groups, respectively. Number of following and grooming episodes, notably after social contact, were normalized in mutant mice trained under the SI-R but not SI-NR condition. Data are presented as mean ± SEM. Animal numbers per genotype, gender and training condition: n=4. Solid stars: genotype x condition interaction (three-way ANOVA followed by Newman-Keules post-hoc test). Two stars p<0.01, three stars p<0.001. OI-R: object interaction – reinforced; SI-NR: Social interaction – non reinforced; SI-R: social interaction – reinforced.

**Legends to Supplementary Tables**

**Table S1. List of primers used for qRT-PCR**

**Table S2. Principal component analysis performed on qRT-PCR data after direct social interaction: component loadings for 3 behavioral parameters and 14 qRT-PCR results in variables' and subjects' spaces**

**Table S3. Statistical analysis: Social interaction and alternation parameters measured in *Oprm1^+/+^* and *Oprm1^-/-^* animals before behavioral training**

**Table S4. Statistical analysis: Social interaction parameters and food intake measured in *Oprm1^+/+^* and *Oprm1^-/-^* animals during the course of behavioral training**

**Table S5. Statistical analysis: Effects of behavioral training conditions on social and non-social behavioral measurements in *Oprm1^+/+^* and *Oprm1^-/-^* animals**

**Table S6: Statistical analysis: Social interaction parameters over repeated testing, up to PN week 24, in *Oprm1^+/+^* and *Oprm1^-/-^* animals of the SI-NR and SI-R groups**

**Table S7. Statistical analysis: Social interaction parameters measured 45 min before qRT-PCR experiment**

**Table S8. Transcription levels of a set of 13 genes across four brain regions in Oprm1-/- versus Oprm1+/+ mice after behavioral therapy**

**References**

1. Crawley JN. Mouse behavioral assays relevant to the symptoms of autism. *Brain pathology* 2007; **17**(4)**:** 448-459.

2. Silverman JL, Yang M, Lord C, Crawley JN. Behavioural phenotyping assays for mouse models of autism. *Nat Rev Neurosci* 2010; **11**(7)**:** 490-502.

3. Moustgaard A, Hau J, Lind NM. Effects of dopamine D4 receptor antagonist on spontaneous alternation in rats. *Behavioral and brain functions : BBF* 2008; **4:** 49.

4. Thomas A, Burant A, Bui N, Graham D, Yuva-Paylor LA, Paylor R. Marble burying reflects a repetitive and perseverative behavior more than novelty-induced anxiety. *Psychopharmacology (Berl)* 2009; **204**(2)**:** 361-373.

5. Meirsman AC, Le Merrer J, Diaz J, Clesse D, Kieffer BL, Becker JAJ. Mice lacking GPR88 show motor deficit, improved spatial learning and low anxiety reversed by delta opioid antagonist. *Submitted to Biological Psychiatry* 2016.

6. Becker JA, Clesse D, Spiegelhalter C, Schwab Y, Le Merrer J, Kieffer BL. Autistic-like syndrome in mu opioid receptor null mice is relieved by facilitated mGluR4 activity. *Neuropsychopharmacology* 2014; **39**(9)**:** 2049-2060.

7. Pobbe RL, Pearson BL, Defensor EB, Bolivar VJ, Young WS, 3rd, Lee HJ*, et al*. Oxytocin receptor knockout mice display deficits in the expression of autism-related behaviors. *Horm Behav* 2012; **61**(3)**:** 436-444.

8. Tabet R, Moutin E, Becker JA, Heintz D, Fouillen L, Flatter E*, et al*. Fragile X Mental Retardation Protein (FMRP) controls diacylglycerol kinase activity in neurons. *Proc Natl Acad Sci U S A* 2016; **113**(26)**:** E3619-3628.

9. Matsuo N, Tanda K, Nakanishi K, Yamasaki N, Toyama K, Takao K*, et al*. Comprehensive behavioral phenotyping of ryanodine receptor type 3 (RyR3) knockout mice: decreased social contact duration in two social interaction tests. *Front Behav Neurosci* 2009; **3:** 3.

10. Katayama Y, Nishiyama M, Shoji H, Ohkawa Y, Kawamura A, Sato T*, et al*. CHD8 haploinsufficiency results in autistic-like phenotypes in mice. *Nature* 2016; **537**(7622)**:** 675-679.

11. Spencer CM, Alekseyenko O, Serysheva E, Yuva-Paylor LA, Paylor R. Altered anxiety-related and social behaviors in the Fmr1 knockout mouse model of fragile X syndrome. *Genes Brain Behav* 2005; **4**(7)**:** 420-430.

12. Becker JA, Kieffer BL, Le Merrer J. Differential behavioral and molecular alterations upon protracted abstinence from cocaine versus morphine, nicotine, THC and alcohol. *Addict Biol* 2017; **22**(5)**:** 1205-1217.

13. Le Marec N, Ethier K, Rompre PP, Godbout R. Involvement of the medial prefrontal cortex in two alternation tasks using different environments. *Brain and cognition* 2002; **48**(2-3)**:** 432-436.

14. Delotterie D, Ruiz G, Brocard J, Schweitzer A, Roucard C, Roche Y*, et al*. Chronic administration of atypical antipsychotics improves behavioral and synaptic defects of STOP null mice. *Psychopharmacology (Berl)* 2010; **208**(1)**:** 131-141.

15. Le Merrer J, Rezai X, Scherrer G, Becker JA, Kieffer BL. Impaired Hippocampus-Dependent and Facilitated Striatum-Dependent Behaviors in Mice Lacking the Delta Opioid Receptor. *Neuropsychopharmacology* 2013.
