# Supplementary material for: Back-translating behavioral intervention for autism spectrum disorders to mice with blunted reward restores social abilities
Source: Transl Psychiatry. 2018 Sep 21;8:197. doi: 10.1038/s41398-018-0247-y (PMC6155047; doi:10.1038/s41398-018-0247-y)
Supplement: Supplementary file 2 — Table S1 [file 41398_2018_247_MOESM2_ESM.pdf]

Table S1. List of primers used for qRT-PCR

RefSeq	Gene name	Gene title	Forward oligonucleotide	Reverse oligonucleotide
NM_007393	actin, beta	Actb	GTATGCCTCGGTCGTACCA	CTTCTGCATCCTGTCAGCAA
NM_018790	activity regulated cytoskeletal-associated protein	*Arc*	CCAGGAGAATGACACCAG	TTCAGGAGAAGAGAGGATG
NM_009732	arginine vasopressin	*Avp*	ACACTACGCTCTTCCGCTTGT	CACTGTCTCAGCTCCATGTCA
NM_016847	arginine vasopressin receptor 1A	*Avpr1a*	GGAGAAACGGGAGACAGACA	AAGCCCATTGTACAGCCCAAG
NM_011924	arginine vasopressin receptor 1B	*Avpr1b*	AGGAGGAAGAGGAGGAGGAA	ACTGAAGGCAGGAACTGAAG
NM_007540	brain derived neurotrophic factor	*Bdnf*	GTGACTGAAAAAGTTCCACC	GACGTTTACTTCTTTCATGGG
NM_205769	corticotropin releasing hormone	*Crh*	AGGAGGCATCCTGAGAGAAGT	ATGTTAGGGGCGCTCTCTTC
NM_010234	FBJ osteosarcoma oncogene	*Fos*	GAAGGGAACGGAATAAGATG	CATCTTCAAGTTGATCTGTCTC
NM_053202	forkhead box P1	*Foxp1*	GCAGCAGCAGCAAGTTAGTG	ATAGCCACTGACACGGGAAC
NM_001013385	glutamate receptor, metabotropic 4	*Grm4*	CTTCCTTAGCCAGGGTCTCC	CATCCCTTCGGACACAGTTT
NM_138666	neuroligin 1	*Nlgn1*	CATTGCATCTTTCTCGCTGA	CCCTCCAGCTGTTGACTCTC
NM_011025	oxytocin	*Oxt*	CTGCTTGGCTTACTGGCTCT	GGGAGACACTTGCGCATATC
NM_001081147	oxytocin receptor	*Oxtr*	CTTAGGGCCAAAAGGTGTCA	GCAGGTTTCTATGCCCTCTG
NM_007475	ribosomal protein, large, P0	*Rplp0*	TTCAATGGTGCCTCTGGAGAT	CATCTTCAAGTTGATCTGTCTC
