# Supplementary material for: Back-translating behavioral intervention for autism spectrum disorders to mice with blunted reward restores social abilities
Source: Transl Psychiatry. 2018 Sep 21;8:197. doi: 10.1038/s41398-018-0247-y (PMC6155047; doi:10.1038/s41398-018-0247-y)
Supplement: Supplementary file 3 — Table S2 [file 41398_2018_247_MOESM3_ESM.pdf]

Table S2. Principal component analysis performed on qRT-PCR data after direct social interaction: component loadings for 3 behavioral parameters and 14 qRT-PCR results in variables' and subjects' spaces

Variable's space

Variables	PC1	PC2	PC3	PC4	PC5	PC6	PC7	PC8	PC9
Time in nose contact (TNC)	**-0,82**	0,04	-0,26	-0,20	-0,40	-0,15	0,06	-0,08	-0,10
Number of following episodes (Following)	**-0,75**	-0,24	-0,38	-0,32	0,27	-0,17	0,07	0,05	0,01
Number of grooming episodes after social contact (GASC)	**0,91**	-0,10	-0,09	-0,01	0,00	-0,26	-0,18	-0,11	-0,05
NAc-*Cfos*	0,01	**0,96**	0,03	0,11	0,04	-0,12	-0,06	0,07	-0,03
NAc-*Oxt*	-0,24	**0,94**	0,06	-0,03	0,04	-0,05	-0,02	-0,06	-0,11
NAc-*Oxtr*	**0,74**	0,05	-0,50	0,36	-0,10	0,16	-0,05	-0,08	0,00
NAc-*Crh*	**-0,74**	-0,46	0,23	0,25	-0,14	-0,22	0,00	0,09	0,06
CeA-*Cfos*	**0,90**	-0,12	0,03	-0,26	0,08	0,15	0,06	-0,08	-0,04
CeA-*Arc*	**0,92**	0,25	0,05	0,03	0,07	-0,09	0,15	0,00	-0,04
CeA-*Oxt*	**-0,92**	0,13	0,02	-0,08	-0,02	0,12	-0,19	-0,18	0,12
CeA-*Avpr1a*	**0,90**	-0,19	-0,07	0,01	0,10	-0,17	-0,20	-0,01	0,15
VTA-*Arc*	**0,94**	0,11	0,04	0,09	0,00	-0,12	0,12	-0,16	0,06
MeA-*Cfos*	**-0,78**	0,55	0,01	-0,09	0,05	0,01	-0,05	-0,05	0,12
MeA-*Arc*	**0,93**	-0,12	0,06	-0,04	0,03	-0,01	-0,09	0,05	-0,21
MeA-*Oxt*	**-0,87**	-0,30	0,09	0,04	0,10	0,04	-0,23	-0,05	-0,21
CPu-*Cfos*	**-0,86**	0,09	-0,24	0,29	0,16	0,00	0,03	0,14	-0,04
CPu-*Grm4*	**-0,84**	-0,24	0,08	0,25	0,18	-0,07	0,15	-0,29	-0,06
% variance explained	65,45	15,86	3,62	3,46	2,10	1,80	1,47	1,29	1,07

Subjects' space

Genotype	Condition	PC1	PC2	PC3	PC4	PC5	PC6	PC7	PC8	PC9
Oprm1 $^{+/+}$	SI-R	**-2,73**	-0,32	-1,12	1,47	-0,38	-0,14	-0,31	-0,06	-0,14
Oprm1 $^{+/+}$	SI-R	**-2,84**	-0,70	1,11	0,17	1,22	-0,18	-0,43	0,31	0,18
Oprm1 $^{+/+}$	SI-R	**-2,87**	-0,21	0,17	0,99	-0,53	-0,27	0,27	0,05	0,12
Oprm1 $^{+/+}$	SI-R	**-2,26**	0,41	**-1,70**	1,12	-0,62	0,15	-0,33	0,51	0,22
Oprm1 $^{+/+}$	SI-R	**-1,99**	-0,55	-0,53	**1,90**	-0,59	1,09	-0,25	-0,09	-0,15
Oprm1 $^{+/+}$	SI-R	**-3,15**	-0,04	1,21	0,98	-0,77	-1,10	-0,03	0,41	0,18
Oprm1 $^{+/+}$	SI-R	**-3,36**	-1,00	**1,69**	0,56	-0,29	**-1,80**	0,00	0,46	0,32
Oprm1 $^{+/+}$	SI-R	**-2,87**	-0,67	0,39	0,41	0,72	-0,69	-0,79	-0,02	-0,01
Oprm1 $^{-/-}$	SI-R	**-1,75**	0,05	-0,08	-1,39	-0,24	-0,07	-0,12	0,16	-0,04
Oprm1 $^{-/-}$	SI-R	**-1,74**	-0,07	0,86	-0,52	-0,91	0,32	-0,06	-0,87	-0,12
Oprm1 $^{-/-}$	SI-R	-0,92	-0,06	0,99	-1,14	0,03	1,03	-0,73	0,26	0,28
Oprm1 $^{-/-}$	SI-R	-0,95	0,21	1,33	-0,69	-0,83	0,24	-0,63	-0,32	0,10
Oprm1 $^{-/-}$	SI-R	**-2,02**	0,49	0,26	-1,28	-1,05	-0,21	0,25	-0,13	0,18
Oprm1 $^{-/-}$	SI-R	**-1,68**	-0,08	0,23	-1,00	-0,21	0,33	-0,16	0,25	0,53
Oprm1 $^{-/-}$	SI-R	**-1,79**	0,20	0,40	-0,58	0,22	0,16	-0,19	0,21	-0,05
Oprm1 $^{-/-}$	SI-R	-1,43	0,03	0,06	-0,52	-0,54	-0,15	-0,61	0,71	0,05
Oprm1 $^{+/+}$	SI-NR	**-2,18**	-0,44	-0,04	0,56	0,17	-0,01	-0,11	-0,09	-0,60
Oprm1 $^{+/+}$	SI-NR	**-1,78**	-0,60	0,07	-0,22	0,24	0,30	0,33	-0,14	-0,39
Oprm1 $^{+/+}$	SI-NR	**-1,84**	-0,12	0,62	-0,01	-0,59	0,41	0,20	-0,22	0,33
Oprm1 $^{+/+}$	SI-NR	**-2,19**	-0,32	-0,14	1,00	-0,04	0,32	0,18	-0,76	0,25
Oprm1 $^{+/+}$	SI-NR	**-1,65**	-0,03	-0,74	-0,28	0,86	0,51	0,31	0,50	-0,18
Oprm1 $^{+/+}$	SI-NR	**-1,55**	-0,04	0,54	0,62	0,12	1,06	-0,31	-0,20	-1,05
Oprm1 $^{+/+}$	SI-NR	**-2,31**	-0,67	0,23	0,43	0,16	0,15	0,10	-0,30	-0,16
Oprm1 $^{+/+}$	SI-NR	**-2,60**	-0,15	0,23	0,22	0,47	-0,06	0,31	0,01	-0,52
Oprm1 $^{-/-}$	SI-NR	**2,97**	**3,02**	0,06	0,38	-0,51	0,44	0,76	-0,02	0,19
Oprm1 $^{-/-}$	SI-NR	**3,42**	**3,35**	0,99	-0,20	-0,35	-0,29	0,66	0,43	-0,28
Oprm1 $^{-/-}$	SI-NR	**2,67**	**3,02**	-0,92	-0,73	-0,31	-0,12	**-1,51**	0,51	-0,88
Oprm1 $^{-/-}$	SI-NR	**2,87**	**3,15**	0,18	0,51	0,79	-0,98	0,15	-1,36	-0,04
Oprm1 $^{-/-}$	SI-NR	**3,31**	**3,91**	-0,01	-0,06	0,25	0,27	0,54	-0,09	0,17
Oprm1 $^{-/-}$	SI-NR	**3,08**	**3,61**	-0,14	0,83	-0,08	-0,30	-0,04	-0,48	0,53
Oprm1 $^{-/-}$	SI-NR	**2,08**	**2,27**	0,19	-0,04	1,25	0,18	0,02	0,02	0,27
Oprm1 $^{-/-}$	SI-NR	**3,61**	**3,26**	-0,77	0,01	-0,04	-0,13	-0,51	0,76	-0,07
Oprm1 $^{+/+}$	OI-R	**-2,27**	-0,55	-0,56	0,00	1,19	0,36	0,13	0,09	0,41
Oprm1 $^{+/+}$	OI-R	**-2,66**	-0,30	-0,91	-0,46	0,32	-0,17	0,15	-0,03	-0,01
Oprm1 $^{+/+}$	OI-R	**-1,80**	-0,38	0,06	0,40	0,81	0,40	0,26	-0,02	0,54
Oprm1 $^{+/+}$	OI-R	**-2,54**	-0,52	-0,82	-0,66	0,48	-0,49	0,36	-0,02	0,15
Oprm1 $^{+/+}$	OI-R	**-2,39**	-0,63	-0,36	-0,39	0,93	0,12	0,17	0,36	0,06
Oprm1 $^{+/+}$	OI-R	**-2,48**	-0,67	-0,13	-0,48	-0,08	0,41	0,11	-0,38	-0,12
Oprm1 $^{+/+}$	OI-R	**-2,87**	-0,50	-0,55	-0,19	-0,16	-0,01	0,24	-0,26	-0,25
Oprm1 $^{+/+}$	OI-R	**-3,47**	-0,51	**-2,30**	**-1,64**	-0,76	-0,88	1,10	-0,39	-0,24
Oprm1 $^{-/-}$	OI-R	**5,97**	**-1,92**	0,65	0,38	0,14	0,28	0,23	-0,02	0,16
Oprm1 $^{-/-}$	OI-R	**6,58**	**-2,07**	-0,10	0,04	-0,37	-0,03	-0,46	-0,41	-0,56
Oprm1 $^{-/-}$	OI-R	**4,45**	-1,33	0,73	-0,24	0,52	0,24	1,11	0,95	-0,18
Oprm1 $^{-/-}$	OI-R	**6,47**	**-2,30**	-0,18	0,38	-0,29	-0,50	0,53	1,07	-0,59
Oprm1 $^{-/-}$	OI-R	**5,79**	**-2,33**	0,82	-1,13	0,28	-0,13	-0,01	-0,95	-0,72
Oprm1 $^{-/-}$	OI-R	**4,96**	**-1,66**	-0,16	-0,12	-0,97	0,96	0,23	0,17	1,25
Oprm1 $^{-/-}$	OI-R	**6,55**	**-2,88**	-0,58	1,17	-0,20	-0,29	0,10	-0,11	-0,14
Oprm1 $^{-/-}$	OI-R	**6,17**	**-2,36**	-1,24	-0,57	0,52	-0,74	-1,17	-0,45	1,00
% variance explained		65,59	15,86	3,60	3,44	2,07	1,77	1,46	1,29	1,06

Component loadings over the absolute value of 0.7 (variable's space) or 1.5 (subjects' space), considered as most significant, are displayed in bold. Percent of total variance explained by each component is shown at the bottom for each condition. Data for PC1 and PC2 are plotted in Figure 5b. CeA: central nucleus of the amygdala, CPu: caudate putamen, NAc: nucleus accumbens, MeA: medial nucleus of the amygdala, VTA: ventral tegmental area; gene names in table S1.
