# Supplementary material for: Back-translating behavioral intervention for autism spectrum disorders to mice with blunted reward restores social abilities
Source: Transl Psychiatry. 2018 Sep 21;8:197. doi: 10.1038/s41398-018-0247-y (PMC6155047; doi:10.1038/s41398-018-0247-y)
Supplement: Supplementary file 4 — Table S3 [file 41398_2018_247_MOESM4_ESM.pdf]

Table S3. Statistical analysis: Social interaction and alternation parameters measured in *Oprm1*$^{+/+}$ and *Oprm1*$^{-/-}$ animals before behavioral training

Oprm1$^{+/+}$	*Oprm1*$^{-/-}$	Assay	Parameter	Genotype effect	Gender effect	Condition effect	Interactions
NoT: 10M, 10F; OI-R: 8M, 8F; SI-NR: 8M, 8F; SI-R: 10M, 9F	NoT: 10M, 10F; OI-R: 8M, 8F; SI-NR: 8M, 8F; SI-R: 9M, 10F	Social interaction	Time spent in nose contact	$F_{1,125}$=379.2, p<0.0001	$F_{1,125}$<1, NS	$F_{3,125}$<1, NS	
			Number of nose contacts	$F_{1,125}$=99.2, p<0.0001	$F_{1,125}$<1, NS	$F_{3,125}$=1.3, NS	
			Mean nose contact duration	$F_{1,125}$=227.0, p<0.0001	$F_{1,125}$=1.2, NS	$F_{3,125}$<1, NS	
			Number of following episodes	$F_{1,125}$=204.3, p<0.0001	$F_{1,125}$<1, NS	$F_{3,125}$=1.2, NS	
			Number of grooming episodes	$F_{1,125}$=21.3, p<0.01	$F_{1,125}$=1.7, NS	$F_{3,125}$<1, NS	
			Grooming after social contact	$F_{1,125}$=252.3, p<0.0001	$F_{1,125}$<1, NS	$F_{3,125}$<1, NS	
		Y-Maze	SPA	$F_{1,125}$=26.2, p<0.0001	$F_{1,125}$=3.0, NS	$F_{3,125}$=2.2, NS	
			AAR	$F_{1,125}$=1.9, NS	$F_{1,125}$=1.4, NS	$F_{1,125}$=1.4, NS	
			SAR	$F_{1,125}$=23.2, p<0.0001	$F_{1,125}$<1, NS	$F_{3,125}$<1, NS	

AAR: alternate arm return; Cond: condition; F: female; OI-R: object interaction - reinforced; M: male; NoT: no training; NS: non significant; SAR: same arm return; SI-NR: social interaction - non reinforced; SI-R: social interaction, reinforced; SPA: spontaneous alternation. See Figure 2.
