# Supplementary material for: Back-translating behavioral intervention for autism spectrum disorders to mice with blunted reward restores social abilities
Source: Transl Psychiatry. 2018 Sep 21;8:197. doi: 10.1038/s41398-018-0247-y (PMC6155047; doi:10.1038/s41398-018-0247-y)
Supplement: Supplementary file 5 — Table S4 [file 41398_2018_247_MOESM5_ESM.pdf]

Table S4. Statistical analysis: Social interaction parameters and food intake measured in *Oprm1* $^{+/+}$ and *Oprm1* $^{-/-}$ animals during the course of behavioral training

Oprm1 $^{+/+}$	*Oprm1* $^{-/-}$	Assay	Parameter	Genotype effect	Gender effect	Condition effect	Interactions	Training effect	Interactions
**OI-R:8M, 8F; SI-R: 10M, 9F**	**OI-R: 8M, 8F; SI-R: 9M, 10F**	**Reinforcement**	Food intake	$F_{1,64}$=10.3, p<0.01	$F_{1,64}$<1, NS	$F_{1,64}$<1, NS		$F_{14,386}$=6.2, p<0.0001	Train x Gender $F_{14,386}$=2.0, p<0.05<br>Train x Geno $F_{14,386}$=2.2, p<0.01<br>Train x Gender x Geno $F_{14,386}$=2.2, p<0.01
**SI-NR: 8M, 8F; SI-R: 10M, 9F**	**SI-NR: 8M, 8F; SI-R: 9M, 10F**	**Social interaction**	Time in nose contact	$F_{1,62}$<1, NS	$F_{1,62}$<1, NS	$F_{1,62}$=12.8, p<0.001	Geno x Cond x Gender $F_{1,62}$=5.5, p<0.05	$F_{4,248}$=12.1, p<0,0001	Train x Cond $F_{4,248}$=3.1, p<0.05
			Number of nose contacts	$F_{1,62}$<1, NS	$F_{1,62}$<1, NS	$F_{1,62}$=6.1, p<0.05	Geno x Cond x Gender $F_{1,70}$=5.0, p<0.05	$F_{4,248}$=5.4, p<0,001	Train x Gender $F_{4,248}$=4.8, p<0.001
			Mean nose contact duration	$F_{1,62}$=10.6, p<0.01	$F_{1,62}$=1.9, NS	$F_{1,62}$=13.0, p<0.001		$F_{4,248}$=15.4, p<0,0001	Train x Cond $F_{4,248}$=2.6, p<0.05

Cond: condition; F: female; Geno: genotype; M: male; NoT: no training; NS: non significant; SI-NR: social interaction - non reinforced; SI-R: social interaction, reinforced; Train: training effect, repeated measures (days 2, 4, 8, 12, 14). See Figures S3 and S4.
