# Supplementary material for: Back-translating behavioral intervention for autism spectrum disorders to mice with blunted reward restores social abilities
Source: Transl Psychiatry. 2018 Sep 21;8:197. doi: 10.1038/s41398-018-0247-y (PMC6155047; doi:10.1038/s41398-018-0247-y)
Supplement: Supplementary file 6 — Table S5 [file 41398_2018_247_MOESM6_ESM.pdf]

Table S5. Statistical analysis: Effects of behavioral training conditions on social and non-social behavioral measurements in *Oprm1*$^{+/+}$ and *Oprm1*$^{-/-}$ animals

Oprm1$^{+/+}$	*Oprm1*$^{-/-}$	Assay	Parameter	Genotype effect	Gender effect	Condition effect	Interactions	Stimulus effect	Interactions	
NoT: 10M, 10F; OI-R: 7M, 8F; SI-NR: 8M, 8F; SI-R: 10M, 9F	NoT: 10M, 10F; OI-R: 8M, 8F; SI-NR: 8M, 8F; SI-R: 9M, 10F	**Social interaction**	Time spent in nose contact	$F_{1,125}$=100.0, p<0.0001	$F_{1,125}$<1, NS	$F_{3,125}$=31.0, p<0.0001	Geno x Cond $F_{3,125}$=28.0, p<0.0001			
			Number of nose contacts	$F_{1,125}$=39.8, p<0.0001	$F_{1,125}$=1.2, NS	$F_{3,125}$=10.7, p<0.0001	Geno x Cond $F_{3,125}$=18.1, p<0.0001			
			Mean duration of nose contacts	$F_{1,125}$=150.5, p<0.0001	$F_{1,125}$<1, NS	$F_{3,125}$=58.6, p<0.0001	Geno x Cond $F_{3,125}$=24.6, p<0.0001			
			Number of following episodes	$F_{1,125}$=103.2, p<0.0001	$F_{1,125}$=4.8, p<0.05	$F_{3,125}$=13.5, p<0.0001	Geno x Cond $F_{3,125}$=18.3, p<0.0001			
			Number of grooming episodes	$F_{1,125}$=5.4, p<0.01	$F_{1,125}$=7.0, p<0.01	$F_{1,125}$=15.9, p<0.0001	Gender x Cond $F_{3,125}$=8.8, p<0.0001<br>Geno x Cond $F_{3,125}$=5.0, p<0.0001			
			Grooming after social contact	$F_{1,125}$=149.5, p<0.0001	$F_{1,125}$=8.2, p<0.01	$F_{3,125}$=44.7, p<0.0001	Gender x Cond $F_{3,125}$=5.2, p<0.01<br>Geno x Cond $F_{3,125}$=40.7, p<0.0001			
NoT: 9M, 10F; OI-R: 7M, 8F; SI-NR: 8M, 8F; SI-R: 10M, 9F	NoT: 9M, 10F; OI-R: 8M, 8F; SI-NR: 8M, 8F; SI-R: 9M, 10F	**3-Chamber test**	Time in compartments	$F_{1,123}$=3.6, NS	$F_{1,123}$<1, NS	$F_{3,123}$=4.6, p<0.01		$F_{1,123}$<1, NS		Habituation phase
			Time spent in close contact	$F_{1,123}$=6.9, p<0.01	$F_{1,123}$<1, NS	$F_{3,123}$=14.3, p<0.0001	Geno x Cond $F_{3,123}$=3.2, p<0.05	$F_{1,123}$<1, NS		
			Number of close contacts	$F_{1,123}$<1, NS	$F_{1,123}$=3.2, NS	$F_{3,123}$=2.7, p<0.05	Geno x Gender $F_{3,125}$=5.6, p<0.05<br>Geno x Gender x Cond $F_{3,125}$=4.4, p<0.05	$F_{1,123}$<1, NS		
			Mean duration of close contacts	$F_{1,123}$=15.2, p<0.001	$F_{1,123}$<1, NS	$F_{3,123}$=22.7, p<0.001	Geno x Cond $F_{3,123}$=6.8, p<0.001	$F_{1,123}$<1, NS		
			Interaction ratio	$F_{1,123}$<1, NS	$F_{1,123}$<1, NS	$F_{3,123}$<1, NS				
			Time in chambers	$F_{1,123}$=5.2, p<0.05	$F_{1,123}$<1, NS	$F_{3,123}$=3.9, p<0.05		$F_{1,123}$=75.5, p<0.0001	Stim x Gender $F_{1,123}$=12.9, p<0.001<br>Stim x Gender x Genot $F_{1,123}$=5.0, p<0.05	Interaction phase
			Time spent in close contact	$F_{1,123}$=1.6, NS	$F_{1,123}$=6.5, p<0.05	$F_{3,123}$=2.9, p<0.05		$F_{1,123}$=212.7, p<0.0001	Stim x Gender $F_{1,123}$=19.7, p<0.0001<br>Stim x Genot $F_{1,123}$=12.1, p<0.001<br>Stim x Cond $F_{1,123}$=3.7, p<0.05	
			Number of close contacts	$F_{1,123}$<1, NS	$F_{1,123}$=3.7, NS	$F_{3,123}$=3.4, p<0.05		$F_{1,123}$=155.2, p<0.0001	Stim x Gender $F_{1,123}$=11.3, p<0.01	
			Mean duration of close contacts	$F_{1,123}$<1, NS	$F_{1,123}$<1, NS	$F_{3,123}$=1.2, NS		$F_{1,123}$=24.1, p<0.0001	Stim x Gender $F_{1,123}$=7.8, p<0.0001<br>Stim x Geno $F_{1,123}$=22.1, p<0.01<br>Stim x Geno x Cond $F_{3,123}$=4.1, p<0.01	
			Interaction ratio	$F_{1,123}$=19.0, p<0.0001	$F_{1,123}$=19.4, p<0.0001	$F_{3,123}$=3.6, p<0.05				
NoT: 10M, 10F; OI-R: 7M, 8F; SI-NR: 8M, 8F; SI-R: 10M, 9F	NoT: 10M, 10F; OI-R: 8M, 8F; SI-NR: 8M, 8F; SI-R: 9M, 10F	**Motor stereotypies**	Rearing	$F_{1,125}$=1.8, NS	$F_{1,125}$<1, NS	$F_{3,125}$=1.1, NS				
			Grooming	$F_{1,125}$=8.1, p<0.01	$F_{1,125}$=9.0, p<0.01	$F_{3,125}$=2.9, p<0.05				
			Burying	$F_{1,125}$=5.7, p<0.05	$F_{1,125}$<1, NS	$F_{3,125}$=1.5, NS				
			Time spent burying	$F_{1,125}$<1, NS	$F_{1,125}$<1, NS	$F_{3,125}$=1.4, NS				
			Burying duration	$F_{1,125}$=12.0, p<0.001	$F_{1,125}$<1, NS	$F_{3,125}$<1, NS				
			Circling	$F_{1,125}$=113.3, p<0.0001	$F_{1,125}$<1, NS	$F_{3,125}$=1.8, NS				
			Head shakes	$F_{1,125}$=30.5, p<0.0001	$F_{1,125}$<1, NS	$F_{3,125}$=2.4, NS				
		**Y-maze**	SPA	$F_{1,125}$=50.1, p<0.0001	$F_{1,125}$=2.4, NS	$F_{3,125}$<1, NS				
			AAR	$F_{1,125}$=1.4, NS	$F_{1,125}$=1.9, NS	$F_{3,125}$=1.9, NS				
			SAR	$F_{1,125}$=104.1, p<0.0001	$F_{1,125}$<1, NS	$F_{3,125}$<1, NS				
		**Marble burying**	Marbles buried	$F_{1,125}$=9.0, p<0.05	$F_{1,125}$<1, NS	$F_{3,125}$=1.7, NS				
		**Novelty-supressed feeding**	Feeding latency	$F_{1,124}$=94.5, p<0.0001	$F_{1,124}$=6.8, p<0.01	$F_{3,124}$=14.2, p<0.0001	Geno x Cond $F_{3,124}$=24.3, p<0.0001<br>Gender x Geno x Cond $F_{3,124}$=2.7, p<0.05			
			Food intake in home cage	$F_{1,124}$=39.4, p<0.0001	$F_{1,124}$=4.9, p<0.05	$F_{3,124}$=8.5, p<0.0001	Geno x Cond $F_{3,124}$=9.0, p<0.0001			

AAR: alternate arm return; Cond: condition; F: female; OI-R: object interaction - reinforced; M: male; NoT: no training; NS: non significant; SAR: same arm return; SI-NR: social interaction - non reinforced; SI-R: social interaction, reinforced; SPA: spontaneous alternation. Stim: stimulus effect, repeated measure (C1 versus C2 or object versus mouse). 2 mice (NoT condition) failed to explore both chambers of the 3-chamber apparatus during the habituation phase and were excluded from the analysis. See Figures 3, 4, S5 and S7.
