# Supplementary material for: Back-translating behavioral intervention for autism spectrum disorders to mice with blunted reward restores social abilities
Source: Transl Psychiatry. 2018 Sep 21;8:197. doi: 10.1038/s41398-018-0247-y (PMC6155047; doi:10.1038/s41398-018-0247-y)
Supplement: Supplementary file 7 — Table S6 [file 41398_2018_247_MOESM7_ESM.pdf]

Table S6. Statistical analysis: Social interaction parameters over repeated testing, up to PN week 24, in *Oprm1*$^{+/+}$ and *Oprm1*$^{-/-}$ animals of the SI-NR and SI-R groups

***Oprm1*$^{+/+}$**	***Oprm1*$^{-/-}$**	**Assay**	**Parameter**	**Genotype effect**	**Gender effect**	**Condition effect**	**Interactions**	**Training effect**	**Interactions Repeated measures**
**SI-NR: 4M, 4F; SI-R: 4M, 5F**	**SI-NR: 4M, 4F; SI-R: 5M, 6F**	**Social interaction**	Time spent in nose contact	$F_{1,28}$=67.5, p<0.0001	$F_{1,28}$<1, NS	$F_{1,28}$=107.1, p<0.0001	Geno x Gend $F_{1,28}$=4.4, p<0.05<br>Geno x Cond $F_{1,28}$=50.2, p<0.0001	$F_{3,84}$=44.4, p<0.0001	Train x Geno $F_{3,84}$=32.6, p<0.0001<br>Train x Cond $F_{3,84}$=30.6, p<0.0001<br>Train x Geno x Cond $F_{3,84}$=13.6, p<0.0001
			Number of nose contacts	$F_{1,28}$=10.4, p<0.01	$F_{1,28}$=1.2, NS	$F_{1,28}$=21.0, p<0.0001	Geno x Cond $F_{1,28}$=12.6, p<0.01<br>Geno x Gender $F_{1,28}$=8.8, p<0.01<br>Cond x Gender $F_{1,28}$=5.8, p<0.05<br>Cond x Geno x Gender $F_{1,28}$=5.7, p<0.05	$F_{3,84}$=40.3, p<0.0001	Train x Geno $F_{3,84}$=7.1, p<0.0001<br>Train x Cond $F_{3,84}$=13.3, p<0.0001<br>Train x Geno x Cond $F_{3,84}$=2.8, p<0.05
			Mean nose contact duration	$F_{1,28}$=153.0, p<0.0001	$F_{1,28}$=2.9, NS	$F_{1,28}$=137.0, p<0.0001	Geno x Cond $F_{1,28}$=48.6, p<0.0001<br>Cond x Gender $F_{1,28}$=5.5, p<0.05<br>Cond x Geno x Gender $F_{1,28}$=4.6, p<0.05	$F_{3,84}$=31.8, p<0.0001	Train x Geno $F_{3,28}$=66.4, p<0.0001<br>Train x Cond $F_{3,84}$=41.3, p<0.0001<br>Train x Geno x Cond $F_{3,84}$=29.8, p<0.0001<br>Train x Geno x Gender x Cond $F_{3,84}$=5.7, p<0.01
			Number of following episodes	$F_{1,28}$=64.7, p<0.0001	$F_{1,28}$<1, NS	$F_{1,28}$=19.3, p<0.001	Geno x Cond $F_{1,28}$=11.2 p<0.01	$F_{3,84}$=5.8, p<0.01	Train x Geno $F_{3,84}$=3.6, p<0.05<br>Train x Cond x Geno $F_{3,84}$=2.8, p<0.05<br>Train x Cond x Gender $F_{3,84}$=3.3, p<0.05
			Grooming	$F_{1,28}$=27.0, p<0.0001	$F_{1,28}$=3.6, NS	$F_{1,28}$<1, NS		$F_{3,84}$=21.7, p<0.0001	Train x Geno $F_{3,84}$=5.2, p<0.05
			Grooming after social contact	$F_{1,28}$=120.1, p<0.001	$F_{1,28}$<1, NS	$F_{1,28}$=16.3, p<0.001	Geno x Cond $F_{1,28}$=6.9, p<0.05	$F_{3,84}$=44.3, p<0.0001	Train x Cond $F_{3,84}$=3.6, p<0.05<br>Train x Geno $F_{3,84}$=43.8, p<0.0001

Cond: condition; F: female; Geno: genotype; M: male; NoT: no training; NS: non significant; SI-NR: social interaction - non reinforced; SI-R: social interaction, reinforced; Train: training effect, repeated measures (tests at PN6, 10, 17 and 24). See Figures 3 and S6.
