# Supplementary material for: Back-translating behavioral intervention for autism spectrum disorders to mice with blunted reward restores social abilities
Source: Transl Psychiatry. 2018 Sep 21;8:197. doi: 10.1038/s41398-018-0247-y (PMC6155047; doi:10.1038/s41398-018-0247-y)
Supplement: Supplementary file 8 — Table S7 [file 41398_2018_247_MOESM8_ESM.pdf]

Table S7. Statistical analysis: Social interaction parameters measured before qRT-PCR experiment

Oprm1$^{+/+}$	*Oprm1*$^{-/-}$	Assay	Parameter	Genotype effect	Gender effect	Condition effect	Interactions
**OB-R: 4 M, 4 F; SI-NR: 4 M, 4 F; SI-R: 4 M, 4 F**	**OB-R: 4 M, 4 F; SI-NR: 4 M, 4 F; SI-R: 4 M, 4 F**	**Social interaction**	Time spent in social contact	$F_{1,36}$=40.1, p<0.0001	$F_{1,36}$<1, NS	$F_{2,36}$=11.7, p<0.001	Geno x Cond $F_{2,36}$=17.5, p<0.0001
			Time spent in nose contact	$F_{1,36}$=35.6, p<0.0001	$F_{1,36}$<1, NS	$F_{2,36}$=9.9, p<0.001	Geno x Cond $F_{2,36}$=16.1, p<0.0001
			Nose contacts	$F_{1,36}$=19.2, p<0.0001	$F_{1,36}$<1, NS	$F_{2,36}$=1.5, NS	Geno x Cond $F_{2,36}$=9.9, p<0.001<br>Gender x Cond $F_{2,36}$=4.0, p<0.05
			Nose contact duration	$F_{1,36}$=32.2, p<0.0001	$F_{1,36}$=2.1, NS	$F_{2,36}$=19.5, p<0.0001	Geno x Cond $F_{2,36}$=12.0, p<0.0001
			Time pent in paw contact	$F_{1,36}$=19.5, p<0.0001	$F_{1,36}$<1, NS	$F_{2,36}$=9.4, p<0.001	Geno x Cond $F_{2,36}$=6.3, p<0.01
			Paw contacts	$F_{1,36}$=24.0, p<0.0001	$F_{1,36}$<1, NS	$F_{2,36}$=9.4, p<0.001	Geno x Cond $F_{2,36}$=7.2, p<0.01
			Paw contact duration	$F_{1,36}$=54.8, p<0.0001	$F_{1,36}$<1, NS	$F_{2,36}$=12.4, p<0.0001	Geno x Cond $F_{2,36}$=13.1, p<0.0001
			Following	$F_{1,36}$=56.1, p<0.0001	$F_{1,36}$<1, NS	$F_{2,36}$=6.5, p<0.01	Geno x Cond $F_{2,36}$=14.7, p<0.0001
			Time spent grooming	$F_{1,36}$=2.7, NS	$F_{1,36}$<1, NS	$F_{2,36}$=4.5, p<0.05	Gender x Geno x Cond $F_{2,36}$=3.5, p<0.05
			Grooming	$F_{1,36}$=8.1, p<0.01	$F_{1,36}$<1, NS	$F_{2,36}$=11.1, p<0.001	
			Grooming duration	$F_{1,36}$<1, NS	$F_{1,36}$<1, NS	$F_{2,36}$<1, NS	
			Grooming after social contact	$F_{1,36}$=72.2, p<0.0001	$F_{1,36}$<1, NS	$F_{2,36}$=21.6, p<0.0001	Geno x Cond $F_{2,36}$=21.8, p<0.0001

Cond: condition; F: female; Geno: genotype; M: male. See Figures 5 and S8.
