# Supplementary material for: Back-translating behavioral intervention for autism spectrum disorders to mice with blunted reward restores social abilities
Source: Transl Psychiatry. 2018 Sep 21;8:197. doi: 10.1038/s41398-018-0247-y (PMC6155047; doi:10.1038/s41398-018-0247-y)
Supplement: Supplementary file 9 — Table S8 [file 41398_2018_247_MOESM9_ESM.pdf]

Table S8. Transcription levels of a set of 13 genes across four brain regions in Oprm1-/- versus Oprm1+/+ mice after behavioral therapy.

				Oprm1$^{-/-}$ OI-R vs *Oprm1*$^{+/+}$ OI-R		*Oprm1*$^{-/-}$ SI-NR vs *Oprm1*$^{+/+}$ OI-R		*Oprm1*$^{-/-}$ SI-R vs *Oprm1*$^{+/+}$ OI-R		*Oprm1*$^{+/+}$ SI-NR vs *Oprm1*$^{+/+}$ OI-R		*Oprm1*$^{+/+}$ SI-R vs *Oprm1*$^{+/+}$ OI-R	
	**RefSeq**	**Gene name**	**Gene title**	**median ± sem**	**p value**	**median ± sem**	**p value**	**median ± sem**	**p value**	**median ± sem**	**p value**	**median ± sem**	**p value**
PFC	NM_007475	ribosomal protein. large. P0	*Rplp0*	1.11 ± 0.09	0.806	-1.12 ± 0.17	0.311	-1.20 ± 0.19	0.948	1,03 ± 0.01	0,461	1.01 ± 0.05	0.540
	NM_010234	FBJ osteosarcoma oncogene	*Fos*	**3.09 ± 0.21**	**0.000**	**3.60 ± 0.10**	**0.000**	3.35 ± 0.18	0.119	**3,49 ± 0.11**	**0.000**	-1.01 ± 0.07	0.119
	NM_018790	activity regulated cytoskeletal-associated protein	*Arc*	**5.80 ± 0.52**	**0.001**	**3.12 ± 0.16**	**0.001**	**5.99 ± 0.28**	**0.000**	**5,01 ± 0.24**	**0.000**	1.03 ± 0.06	0.359
	NM_007540	brain derived neurotrophic factor	*Bdnf*	1.11 ± 0.03	0.701	-1.07 ± 0.11	0.133	1.01 ± 0.11	0.143	1.01 ± 0.05	0,209	1.06 ± 0.05	0.143
	NM_011025	oxytocin	*Oxt*	n.d.		n.d.		n.d.		n.d.		n.d.	
	NM_001081147	oxytocin receptor	*Oxtr*	n.d.		n.d.		n.d.		n.d.		n.d.	
	NM_009732	arginine vasopressin	*Avp*	n.d.		n.d.		n.d.		n.d.		n.d.	
	NM_016847	arginine vasopressin receptor 1A	*Avpr1a*	n.d.		n.d.		n.d.		n.d.		n.d.	
	NM_011924	arginine vasopressin receptor 1B	*Avpr1b*	n.d.		n.d.		n.d.		n.d.		n.d.	
	NM_001013385	glutamate receptor. metabotropic 4	*Grm4*	n.d.		n.d.		n.d.		n.d.		n.d.	
	NM_205769	corticotropin releasing hormone	*Crh*	**3.74 ± 0.27**	**0.001**	**3.36 ± 0.11**	**0.000**	**3.40 ± 0.55**	**0.000**	**2,29 ± 0.24**	**0,041**	1.06 ± 0.05	0.574
	NM_138666	neuroligin 1	*Nlgn1*	1.14 ± 0.13	0.143	1.03 ± 0.08	0.233	-1.11 ± 0.09	0.559	1,08 ± 0.16	0,132	1.10 ± 0.07	0.559
	NM_053202	forkhead box P1	*Foxp1*	**2.13 ± 0.15**	**0.007**	**2.07 ± 0.06**	**0.000**	1.84 ± 0.20	0.859	1,02 ± 0.11	0,289	1.01 ± 0.09	0.859
CPU	NM_007475	ribosomal protein. large. P0	*Rplp0*	1.01 ± 0.04	0.488	1.02 ± 0.04	0.222	1.03 ± 0.01	0.835	1.13 ± 0.04	0.874	-1.07 ± 0.18	0.417
	NM_010234	FBJ osteosarcoma oncogene	*Fos*	**-3.53 ± 0.24**	**0.000**	**-2.33 ± 0.10**	**0.000**	**-2.12 ± 0.18**	**0.000**	1.04 ± 0.09	0.326	1.07 ± 0.07	0.350
	NM_018790	activity regulated cytoskeletal-associated protein	*Arc*	**7.27 ± 0.33**	**0.000**	**5.96 ± 0.52**	**0.000**	**5.83 ± 0.53**	**0.000**	1.00 ± 0.04	0.389	1.02 ± 0.12	0.219
	NM_007540	brain derived neurotrophic factor	*Bdnf*	1.09 ± 0.08	0.565	1.01 ± 0.52	0.245	1.33 ± 0.14	0.067	1.13 ± 0.16	0.070	-1.02 ± 0.11	0.952
	NM_011025	oxytocin	*Oxt*	1.04 ± 0.04	0.684	-1.05 ± 0.13	0.234	-1.15 ± 0.07	0.456	1.04 ± 0.05	0.477	1.07 ± 0.08	0.261
	NM_001081147	oxytocin receptor	*Oxtr*	1.14 ± 0.13	0.629	1.04 ± 0.03	0.001	1.08 ± 0.10	0.231	-1.02 ± 0.07	0.241	1.04 ± 0.18	0.673
	NM_009732	arginine vasopressin	*Avp*	1.15 ± 0.11	0.313	-1.06 ± 0.12	0.245	-1.25 ± 0.08	0.666	-1.02 ± 0.09	0.697	-1.03 ± 0.11	0.128
	NM_016847	arginine vasopressin receptor 1A	*Avpr1a*	**1.62 ± 0.10**	**0.040**	1.15 ± 0.09	0.231	1.06 ± 0.13	0.123	1.05 ± 0.10	0.128	1.01 ± 0.12	0.412
	NM_011924	arginine vasopressin receptor 1B	*Avpr1b*	1.10 ± 0.14	**0.001**	-1.02 ± 0.12	0.000	1.05 ± 0.11	0.129	1.17 ± 0.08	0.135	1.05 ± 0.10	0.413
	NM_001013385	glutamate receptor. metabotropic 4	*Grm4*	**-3.46 ± 0.22**	**0.001**	**-3.37 ± 0.36**	**0.000**	**-2.22 ± 0.18**	**0.000**	-1.01 ± 0.17	0.246	1.17 ± 0.16	0.143
	NM_205769	corticotropin releasing hormone	*Crh*	-1.83 ± 0.10	0.772	-1.43 ± 0.04	0.332	-1.37 ± 0.18	0.139	1.10 ± 0.14	0.145	1.05 ± 0.15	0.356
	NM_138666	neuroligin 1	*Nlgn1*	1.11 ± 0.10	0.811	1.04 ± 0.14	0.342	-1.04 ± 0.12	0.734	-1.03 ± 0.16	0.768	-1.07 ± 0.18	0.615
	NM_053202	forkhead box P1	*Foxp1*	**1.89 ± 0.25**	**0.003**	**1.46 ± 0.12**	**0.004**	1.48 ± 0.18	0.083	1.05 ± 0.19	0.086	1.03 ± 0.04	0.694
NAC	NM_007475	ribosomal protein. large. P0	*Rplp0*	1.01 ± 0.21	0.885	1.03 ± 0.02	0.233	1.01 ± 0.21	0.589	1.20 ± 0.04	0.324	-1.04 ± 0.27	0.940
	NM_010234	FBJ osteosarcoma oncogene	*Fos*	**-1.80 ± 0.09**	**0.003**	**3.23 ± 0.04**	**0.000**	**1.26 ± 0.06**	**0.018**	-1.01 ± 0.07	0.144	**1.47 ± 0.17**	**0.026**
	NM_018790	activity regulated cytoskeletal-associated protein	*Arc*	**6.30 ± 0.35**	**0.000**	**8.19 ± 0.16**	**0.000**	**6.08 ± 0.89**	**0.001**	**2.48 ± 0.44**	**0.014**	**1.80 ± 0.21**	**0.024**
	NM_007540	brain derived neurotrophic factor	*Bdnf*	1.14 ± 0.12	0.676	1.02 ± 0.02	0.000	**3.91 ± 0.42**	**0.000**	**4.20 ± 0.23**	**0.000**	**1.63 ± 0.10**	**0.000**
	NM_011025	oxytocin	*Oxt*	**-4.72 ± 0.17**	**0.000**	**5.21 ± 0.37**	**0.004**	**1.93 ± 0.27**	**0.000**	**1.41 ± 0.19**	**0.016**	1.06 ± 0.08	0.498
	NM_001081147	oxytocin receptor	*Oxtr*	**1.95 ± 0.13**	**0.000**	**1.71 ± 0.10**	**0.008**	-1.27 ± 0.03	0.425	1.04 ± 0.06	0.831	1.09 ± 0.29	0.395
	NM_009732	arginine vasopressin	*Avp*	1.01 ± 0.20	0.284	1.00 ± 0.10	0.244	**4.36 ± 0.33**	**0.003**	1.81 ± 0.13	0.249	**3.87 ± 0.56**	**0.040**
	NM_016847	arginine vasopressin receptor 1A	*Avpr1a*	**-2.07 ± 0.30**	**0.000**	-1.53 ± 0.14	0.501	1.09 ± 0.41	0.138	1.28 ± 0.34	0.088	1.08 ± 0.05	0.799
	NM_011924	arginine vasopressin receptor 1B	*Avpr1b*	1.08 ± 0.13	0.001	1.04 ± 0.07	0.399	-1.09 ± 0.22	0.244	1.02 ± 0.05	0.146	**1.67 ± 0.18**	**0.012**
	NM_001013385	glutamate receptor. metabotropic 4	*Grm4*	**-2.13 ± .26**	**0.004**	**-4.09 ± 0.37**	**0.000**	**-1.68 ± 0.25**	**0.034**	**-1.81 ± 0.13**	**0.016**	1.09 ± 0.09	0.389
	NM_205769	corticotropin releasing hormone	*Crh*	**-2.73 ± 0.14**	**0.005**	**-4.13 ± 0.20**	**0.001**	**-1.20 ± 0.06**	**0.000**	1.16 ± 0.33	0.787	**2.85 ± 0.47**	**0.000**
	NM_138666	neuroligin 1	*Nlgn1*	**-1.57 ± 0.18**	**0.000**	**-1.51 ± 0.11**	**0.000**	**-2.84 ± 0.45**	**0.042**	**1.39 ± 0.08**	**0.002**	**-1.85 ± 0.17**	**0.004**
	NM_053202	forkhead box P1	*Foxp1*	1.01 ± 0.03	0.666	1.00 ± 0.06	0.415	**-1.64 ± 0.14**	**0.004**	1.23 ± 0.58	0.082	1.14 ± 0.33	0.202
MeA	NM_007475	ribosomal protein. large. P0	*Rplp0*	1.12 ± 0.04	0.505	1.03 ± 0.02	0.831	-1.04 ± 0.14	0.312	-1.09 ± 0.13	0.234	1.04 ± 0.06	0.410
	NM_010234	FBJ osteosarcoma oncogene	*Fos*	**-4.07 ± 0.35**	**0.000**	-1.09 ± 0.03	0.063	-1.09 ± 0.05	0.213	-1.04 ± 0.14	0.240	-1.01 ± 0.10	0.119
	NM_018790	activity regulated cytoskeletal-associated protein	*Arc*	**5.63 ± 0.34**	**0.000**	**3.58 ± 0.30**	**0.000**	1.49 ± 0.14	0.099	**1.79 ± 0.25**	**0.047**	**1.24 ± 0.10**	**0.042**
	NM_007540	brain derived neurotrophic factor	*Bdnf*	1.10 ± 0.07	0.106	1.08 ± 0.08	0.488	-1.07 ± 0.14	0.312	1.02 ± 0.07	0.921	1.07 ± 0.12	0.778
	NM_011025	oxytocin	*Oxt*	**-2.40 ± 0.10**	**0.005**	**-2.55 ± 0.15**	**0.000**	1.04 ± 0.10	0.266	1.24 ± 0.12	0.231	1.27 ± 0.12	0.888
	NM_001081147	oxytocin receptor	*Oxtr*	**1.76 ± 0.10**	**0.013**	**-2.45 ± 0.16**	**0.000**	-1.02 ± 0.09	0.473	-1.12 ± 0.14	0.421	1.05 ± 0.08	0.312
	NM_009732	arginine vasopressin	*Avp*	n.d.		n.d.		n.d.		n.d.		n.d.	
	NM_016847	arginine vasopressin receptor 1A	*Avpr1a*	**-1.62 ± 0.11**	**0.051**	**-2.34 ± 0.15**	**0.000**	1.01 ± 0.05	0.862	-1.05 ± 0.05	0.932	1.05 ± 0.18	0.403
	NM_011924	arginine vasopressin receptor 1B	*Avpr1b*	**-1.67 ± 0.15**	**0.008**	**-2.31 ± 0.29**	**0.004**	1.11 ± 0.15	0.349	1.01 ± 0.15	0.135	1.03 ± 0.07	0.671
	NM_001013385	glutamate receptor. metabotropic 4	*Grm4*	n.d.		n.d.		n.d.		n.d.		n.d.	
	NM_205769	corticotropin releasing hormone	*Crh*	1.01 ± 0.05	0.628	-1.12 ± 0.06	0.064	-1.07 ± 0.23	0.635	1.04 ± 0.09	0.241	-1.04 ± 0.21	0.423
	NM_138666	neuroligin 1	*Nlgn1*	1.04 ± 0.08	0.587	-1.10 ± 0.16	0.293	-1.08 ± 0.12	0.126	1.11 ± 0.13	0.412	1.03 ± 0.17	0.711
	NM_053202	forkhead box P1	*Foxp1*	1.02 ± 0.05	0.145	-1.02 ± 0.10	0.497	-1.02 ± 0.08	0.168	1.08 ± 0.10	0.111	1.08 ± 0.14	0.623
CeA	NM_007475	ribosomal protein. large. P0	*Rplp0*	1.11 ± 0.09	0.884	-1.12 ± 0.17	0.231	-1,20 ± 0.19	0,341	1.03 ± 0.01	0.229	1.01 ± 0.05	0.291
	NM_010234	FBJ osteosarcoma oncogene	*Fos*	**2.46 ± 0.08**	**0.000**	**1.67 ± 0.08**	**0.000**	1,13 ± 0.13	0,811	-1.00 ± 0.07	0.759	**-1.58 ± 0.10**	**0.015**
	NM_018790	activity regulated cytoskeletal-associated protein	*Arc*	**7.35 ± 0.42**	**0.000**	**7.43 ± 0.51**	**0.000**	**1,54 ± 0.11**	**0,007**	**2.05 ± 0.15**	**0.000**	1.06 ± 0.16	0.676
	NM_007540	brain derived neurotrophic factor	*Bdnf*	**-1.63 ± 0.06**	**0.000**	**-2.34 ± 0.17**	**0.003**	1,24 ± 0.17	0,177	-1.02 ± 0.11	0.469	1.01 ± 0.08	0.247
	NM_011025	oxytocin	*Oxt*	**-5.31 ± 0.48**	**0.008**	**-3.13 ± 0.20**	**0.000**	1,10 ± 0.09	0,888	-1.01 ± 0.14	0.213	1.05 ± 0.08	0.291
	NM_001081147	oxytocin receptor	*Oxtr*	**1.66 ± 0.08**	**0.000**	**1.43 ± 0.07**	**0.047**	-1,04 ± 0.09	0,612	-1.02 ± 0.12	0.409	1.11 ± 0.25	0.488
	NM_009732	arginine vasopressin	*Avp*	n.d.		n.d.		n.d.		n.d.		n.d.	
	NM_016847	arginine vasopressin receptor 1A	*Avpr1a*	**3.48 ± 0.26**	**0.000**	**2.18 ± 0.15**	**0.000**	1,27 ± 0.11	0,052	-1.07 ± 0.07	0.411	1.11 ± 0.20	0.133
	NM_011924	arginine vasopressin receptor 1B	*Avpr1b*	-1.01 ± 0.07	0.120	-1.05 ± 0.07	0.341	1,16 ± 0.19	0,120	1.63 ± 0.54	0.783	1.14 ± 0.20	0.801
	NM_001013385	glutamate receptor. metabotropic 4	*Grm4*	n.d.		n.d.		n.d.		n.d.		n.d.	
	NM_205769	corticotropin releasing hormone	*Crh*	**2.44 ± 0.20**	**0.000**	**2.97 ± 0.19**	**0.001**	-1,0 ± 0.10	0,716	-1.01 ± 0.04	0.344	1.04 ± 0.10	0.795
	NM_138666	neuroligin 1	*Nlgn1*	**1.60 ± 0.10**	**0.003**	1.19 ± 0.13	0.022	**1,37 ± 0.06**	**0,011**	**1.27 ± 0.02**	**0.005**	-1.01 ± 0.25	0.232
	NM_053202	forkhead box P1	*Foxp1*	**1.39 ± 0.06**	**0.000**	**1.57 ± 0.11**	**0.021**	1,07 ± 0.13	0,126	-1.11 ± 0.13	0.342	1.23 ± 0.32	0.958
VTA	NM_007475	ribosomal protein. large. P0	*Rplp0*	-1.07 ± 0.18	0.417	-1.18 ± 0.19	0.242	1.11 ± 0.08	0.523	-1.07 ± 0.12	0.234	-1.02 ± 0.03	0.325
	NM_010234	FBJ osteosarcoma oncogene	*Fos*	**-2.57 ± 0.25**	**0.000**	-2.70 ± 0.85	0.213	1.12 ± 0.10	0.623	-1.21 ± 0.13	0.623	1.14 ± 0.08	0.676
	NM_018790	activity regulated cytoskeletal-associated protein	*Arc*	**4.87 ± 0.19**	**0.000**	**4.11 ± 0.41**	**0.000**	**1.40 ± 0.08**	**0.002**	**1.46 ± 0.46**	**0.001**	-1.07 ± 0.08	0.572
	NM_007540	brain derived neurotrophic factor	*Bdnf*	-1.03 ± 0.05	0.615	1.02 ± 0.04	0.241	1.09 ± 0.07	0.080	**2.39 ± 0.19**	**0.029**	-1.08 ± 0.11	0.367
	NM_011025	oxytocin	*Oxt*	1.01 ± 0.07	0.673	1.01 ± 0.06	0.342	1.01 ± 0.05	0.87	1.35 ± 0.03	0.123	1.22 ± 0.11	0.782
	NM_001081147	oxytocin receptor	*Oxtr*	-1.09 ± 0.07	0.261	1.07 ± 0.08	0.245	1.10 ± 0.08	0.254	1.07 ± 0.16	0.313	1.13 ± 0.20	0.145
	NM_009732	arginine vasopressin	*Avp*	n.d.		n.d.		n.d.		n.d.		n.d.	
	NM_016847	arginine vasopressin receptor 1A	*Avpr1a*	1.10 ± 0.05	0.128	1.03 ± 0.06	0.241	1.03 ± 0.04	0.531	-1.25 ± 0.16	0.351	1.08 ± 0.04	0.379
	NM_011924	arginine vasopressin receptor 1B	*Avpr1b*	-1.04 ± 0.08	0.412	1.05 ± 0.08	0.612	1.02 ± 0.06	0.237	1.22 ± 0.17	0.234	1.05 ± 0.05	0.462
	NM_001013385	glutamate receptor. metabotropic 4	*Grm4*	n.d.		n.d.		n.d.		n.d.		n.d.	
	NM_205769	corticotropin releasing hormone	*Crh*	**2.46 ± 0.14**	**0.008**	**4.38 ± 0.47**	**0.000**	1.01 ± 0.04	0.264	1.12 ± 0.08	0.341	1.06 ± 0.06	0.412
	NM_138666	neuroligin 1	*Nlgn1*	1.01 ± 0.12	0.694	-1.01 ± 0.07	0.134	-1.01 ± 0.12	0.452	-1.10 ± 0.17	0.122	1.04 ± 0.06	0.612
	NM_053202	forkhead box P1	*Foxp1*	1.03 ± 0.07	0.219	1.20 ± 0.11	0.245	-1.01 ± 0.09	0.386	1.21 ± 0.23	0.386	1.02 ± 0.07	0.476

Data are expressed as fold change versus the *Oprm1*$^{+/+}$ OI-R group (median ± SEM). Student's t-tests were performed on transformed data (see Material and Methods) to determine whether fold changes differed from 0 (no regulation: corresponds to ± 1 in table). Significant regulations with a fold change over ± 1.20 are highlighted in bold (considered biologically significant). Boxes are filled in red for significant up-regulation of gene expression, in blue for significant down-regulation. n.d.: not detected.
